# *Bmal1* deficiency in neutrophils alleviates symptoms induced by high-fat diet

**DOI:** 10.1016/j.isci.2025.112038

**Published:** 2025-02-25

**Authors:** Brinja Leinweber, Violetta Pilorz, Iwona Olejniczak, Ludmila Skrum, Kimberly Begemann, Isabel Heyde, Sarah Stenger, Christian David Sadik, Henrik Oster

**Affiliations:** 1 University of Lübeck, Institute of Neurobiology, Center of Brain, Behaviour and Metabolism, Marie-Curie-Strasse, 23562 Luebeck, Germany; 2 University of Lübeck, Department of Dermatology, Allergy, and Venereology Ratzeburger Allee, 23562 Luebeck, Germany

**Keywords:** Molecular physiology, Immunology, Cell biology, Diet

## Abstract

Physiological processes, including metabolism and immune responses, are generated by the circadian clock, driven by clock genes. Disrupting circadian rhythms through a high-fat diet promotes obesity and inflammation. Studies show that deleting the clock gene, brain, and muscle ARNT-like 1 (*Bmal1*) in adipose tissue leads to overeating and weight gain. We now show that *Bmal1* deletion in neutrophils protects against diet-induced obesity and reduces inflammatory macrophage infiltration into epididymal white adipose tissue (eWAT), despite increased food intake over 20 weeks of a high-fat diet. This protection is linked to enhanced energy expenditure, increased UCP1 expression in iBAT, improved insulin sensitivity, and altered expression of genes encoding chemokine receptors CXCR2, CXCR4, and the ligand Cxcl2 in eWAT. Our findings reveal a key role of *Bmal1* in neutrophils in regulating high-fat diet-induced adipose inflammation and emphasize circadian regulation’s importance in immuno-metabolic function.

## Highlights

• *Bmal1* deficiency in neutrophils (NE-BKO) increases insulin sensitivity on HFD
• NE-BKO mice show reduced energy expenditure with elevated UCP1 in iBAT during HFD
• Increased type 2 macrophage infiltration in eWAT observed in NE-BKO mice on HFD
• NE-BKO mice show high *Cxcr4* and low *Cxcr2* and *Cxcl2* expression in eWAT after HFD

## Graphical abstract

**Figure:**
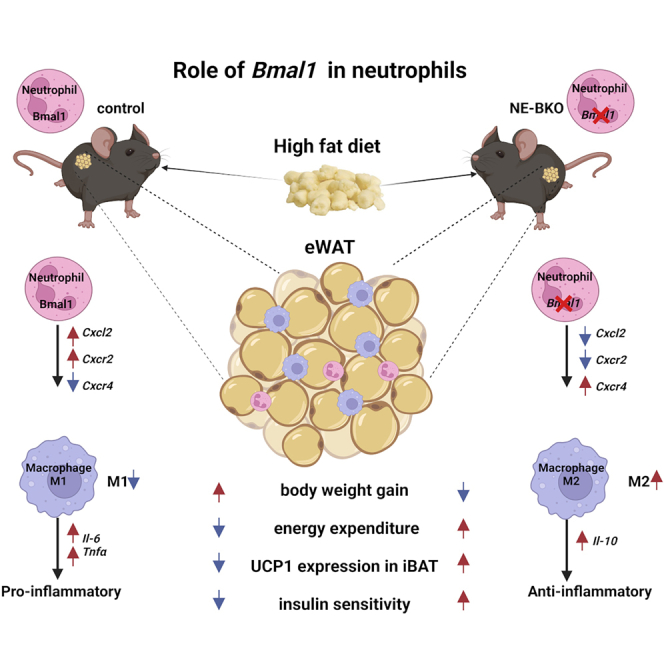

## Introduction

Neutrophils are the most abundant leukocytes in the mammalian system and serve as frontline defenders against infections.^1^ Their distinctive features, marked by granules containing antimicrobial proteins, proteases, and oxidases, as well as the production of cytokines and chemokines, and the formation of neutrophil extracellular traps (NET) qualify neutrophils to act as mediators of anti-microbial defense and tissue inflammation.^2^^,^^3^^,^^4^ The activity of both these processes is timely regulated by neutrophil intrinsic programs across the day.^5^ Neutrophils also play a role in inflammatory responses associated with obesity, characterized by the accumulation of pro-inflammatory macrophages and an increased presence of neutrophils in metabolically active tissues, such as adipose tissue.^6^ This metabolic inflammation leads to increased adipogenesis and lipid storage, and impairment of the ability of adipocytes to release free fatty acids into the circulation, which generally serves as an energy source.^7^

The intricate interplay between neutrophils and macrophages plays a crucial role in influencing the metabolic dynamics in tissues. Both cell types release cytokine signals, such as tumor necrosis factor alpha (TNFα) and interleukin 1 (IL-1), which act as reciprocal signals for further tissue infiltration.^8^ The regulation of neutrophil infiltration is also influenced by intrinsic circadian clocks, which consist of clock genes/proteins forming a set of interlocked transcriptional and translational feedback loops. These feedback loops generate the rhythmic expression of clock genes themselves and, through tissue-specific outputs, coordinate physiological and behavioral rhythms at ∼24-h periodicity.^9^ Consequently, various metabolic events, including glucose metabolism (such as insulin sensitivity and glucose tolerance) and lipid metabolism, exhibit circadian variations.^10^ The intimate connection between the circadian clock and metabolism has been demonstrated in clock gene-deficient mice. For instance, adipocyte-specific *Bmal1-null* mice display increased food intake during the light phase and elevated body weight.^11^ Vice versa, dietary changes, such as a high-fat diet, can disrupt circadian rhythms, leading to a shift in feeding behavior to the light phase, severe body weight changes, insulin intolerance, and disruption in *Bmal1* gene expression rhythms.^12^ Interestingly, recent studies demonstrated contrasting effects. For instance, ***Bmal1****-null* mice exposed to chronic HFD conditions consistently show reduced body weight gain and sustained insulin sensitivity.^13^^,^^14^ These findings indicate a protective mechanism against HFD-induced obesity and metabolic disorders, underscoring the complexity of the relationship between circadian regulation and metabolism.

BMAL1, a component of the clock, induces the rhythmic expression of C-X-C motif chemokine ligand 2 (Cxcl2). Cxcl2 is a ligand of the rhythmically expressed CXCL receptor 2 (CXCR2) on neutrophils, which initiates neutrophil aging, while CXCR4 acts as an antagonist to this process.^5^^,^^15^^,^^16^^,^^17^ Circadian aspects of neutrophil aging have been confirmed by studies using mice with the neutrophil-specific deletion of *Bmal1*.^18^ Neutrophils of these mutants have characteristics of fresh neutrophils, which predominantly migrate into tissues during the night. On the other hand, neutrophil-specific CXCR4 mutants have cells that resemble aged neutrophils, which are usually mostly observed during the day.^5^^,^^18^ This dynamic ensures that neutrophil aging aligns with the body’s circadian rhythm. However, when the balance between CXCR2 and CXCR4 is disrupted, as shown by Adrover et al. 2019^18^ and by Yildirim et al. 2005^19^ an accumulation of senescent neutrophils can occur. These aged neutrophils become retained in the bone marrow or the vasculature, where their presence disrupts normal neutrophil clearance and homeostasis. This retention has been shown to contribute to prolonged inflammation, particularly within the vascular system, highlighting the critical role of circadian regulation in maintaining immune balance and preventing inflammatory disorders.^19^ In conclusion, internal circadian timers orchestrate the neutrophil aging process, thus contributing to the overall dynamics of leukocyte extravasation. They play a critical role in maintaining the delicate balance between antimicrobial defense and tissue inflammation, highlighting the multifaceted impact of neutrophils on tissue homeostasis.

A further crucial feature of neutrophils is their short lifespan. The estimated half-life of neutrophils in circulation is ca. 10 h.^20^ Pathologic environments can influence the lifespan of neutrophils.^21^^,^^22^ Such environments include the presence of other inflammatory immune cells e.g., macrophages. M1 macrophages, as pro-inflammatory cells, extend the survival of neutrophils through signals such as nitric oxide, TNFα, and IL-6.^23^^,^^24^ Additionally, M1-derived pro-inflammatory factors can inhibit adipogenesis.^7^ The interaction between neutrophils and M1 macrophages plays a pivotal role in enhancing the immune response against pathogens with the potential to escalate into overt inflammation and pathology.^8^ In contrast, senescent neutrophils undergo apoptosis and are efficiently cleared by M2 macrophages, which also promote tissue healing. The polarization of macrophages toward the anti-inflammatory M2 phenotype is initiated through the secretion of annexin A1 (ANXA1) by neutrophils, facilitating their own effective clearance through phagocytosis.^25^^,^^26^^,^^27^

Consumption of a high-fat diet has been linked to the promotion of chronic low-grade inflammation within adipose tissue.^28^ This metabolic inflammation is a key factor in the progression of obesity-related pathological conditions, in particular type 2 diabetes.^29^^,^^30^ Among inflammatory cells within adipose tissue, macrophages play a critical role in modulating adipose tissue activity during metabolic inflammation^31^ with macrophage-derived inflammatory markers affecting the expression of adipose genes associated with lipid metabolism.^32^ In addition, neutrophils can influence macrophage phenotypes by promoting a pro-inflammatory M1 phenotype through the release of azurocidin^8^ or by inducing an anti-inflammatory M2 phenotype through the release of ANXA1, thus facilitating neutrophil clearance from adipose stores.^33^^,^^34^

Given that macrophage polarization in response to metabolic changes may depend on the stage of neutrophil senescence, circadian clocks are likely involved in regulating this process. Macrophages, in turn, regulate neutrophil survival and clearance through both inflammatory and anti-inflammatory signaling pathways. Disruptions in neutrophil circadian clocks, particularly through the loss of *Bmal1*, could destabilize this balance by altering the expression of chemokines such as CXCL2 and its receptor CXCR2, which are involved in regulating neutrophil aging and migration. These changes in chemokine signaling could modify macrophage phenotypes, leading to increased metabolic inflammation in adipose tissues. In this study, we aimed at investigating whether neutrophil clocks, particularly ***Bmal1***, influence the development of metabolic inflammation mediators, focusing on the regulation of chemokines and macrophage polarization in adipose tissue. By examining these interactions, we hoped to gain insights into the mechanisms driving metabolic inflammation during chronic high-fat diet conditions.

## Results

### Brain and muscle Arnt-like protein-1 deficiency in neutrophils affects food intake and body mass in mice when fed with a high-fat diet

Consumption of an HFD has been associated with increased body weight and disruption of molecular clock rhythms in peripheral tissues responsible for regulating metabolic functions in mice.^35^ In the long term, these tissues – e.g., eWAT and scWAT – often exhibit inflammation due to an innate immune response.^36^ The current study aimed to explore the role of the neutrophil clock gene *Bmal1* in regulating the pro-inflammatory process induced by HFD.

**Figure 1:**
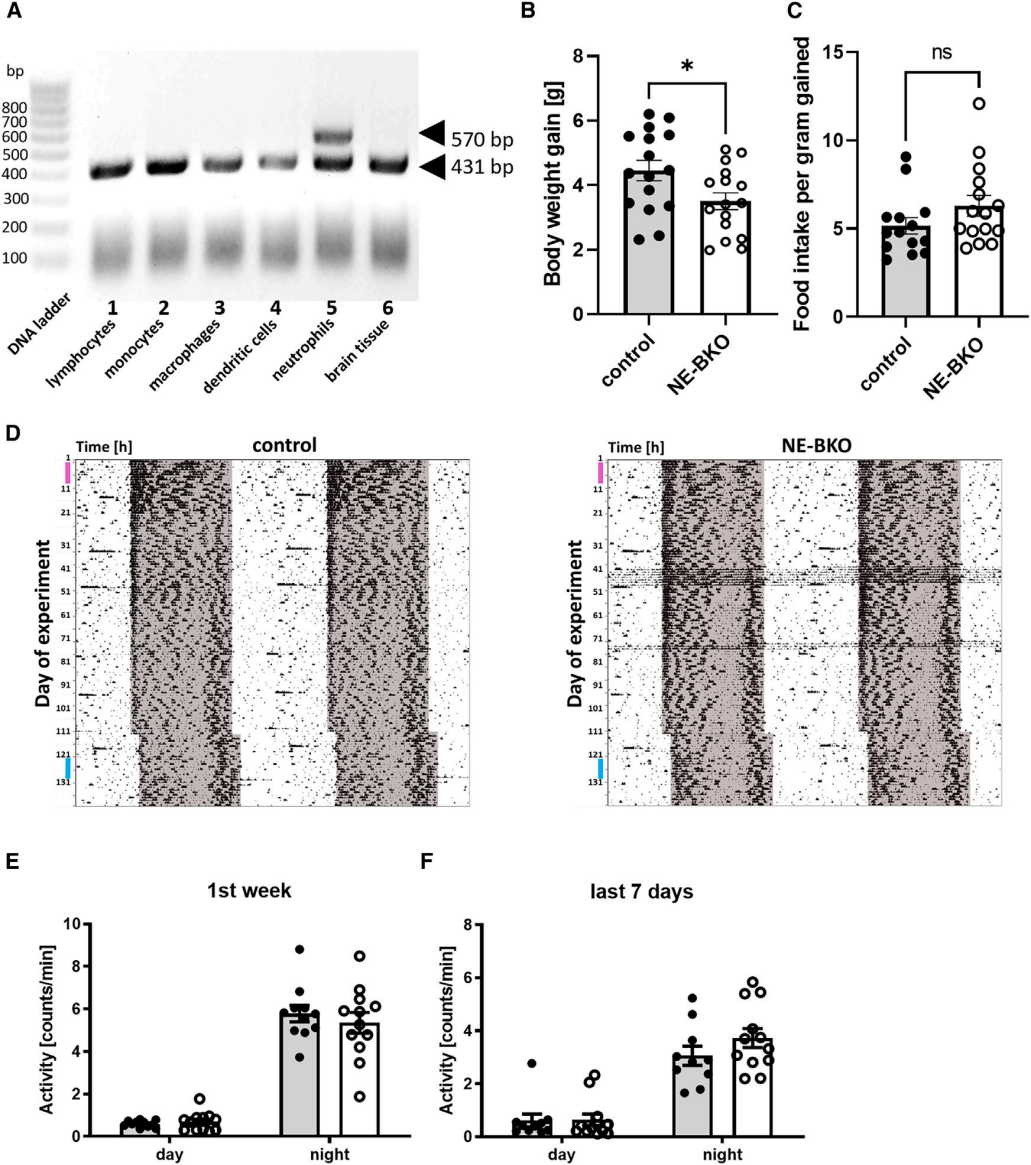
Impact of *Bmal1* deficiency on wheel-running activity and food intake in the first 7 days during HFD (A) PCR products amplified from the following cell types and tissues of an NE-BKO mouse are shown (left to right): 1. lymphocytes (liver), 2. monocytes (bone marrow), 3. macrophages (spleen), 4. dendritic cells (spleen), 5. neutrophils (bone marrow), and 6. brain tissue (cerebellum). Only neutrophils exhibit evidence of homozygous conditional *Bmal1* deletion mediated by active *Mrp8-Cre* recombinase. This is indicated by the presence of two bands: a 431 bp band corresponding to the *Bmal1-flox* allele and a 570 bp band corresponding to the *Bmal1-KO* allele. In contrast, all other immune cells and tissues display only the 431 bp band, indicating the presence of the *Bmal1-flox* allele without *Mrp8-Cre*-mediated recombination. On the left-hand site 100bp ladder. (B) Despite similar food intake per gram of body weight gained during the first week of HFD (t-test, t = 1.52, df = 25.99, *p* = 0.139, control *n* = 16, NE-BKO *n* = 16) (C), NE-BKO mice gained significantly less body weight compared to control mice during this 7-day period (t-test, t = 2.33, df = 29.01, *p* = 0.027, control *n* = 16, NE-BKO *n* = 16). (D) Exemplary actograms show the rhythm of wheel-running activity in both control and NE-BKO mice over a period of 135 days on a high-fat diet (HFD). The HFD did not affect the total amount of wheel-running activity during the 1^st^ 7 days of HFD (E). In addition, there were no differences in wheel-running activity between day and night in the last week of HFD (control *n* = 11, NE-BKO *n* = 12) (F). The shaded area represents the period of darkness. The pink line on the y axis indicates the 7-day period of the first week, while the blue line on the y axis indicates the representative 7-day period of the last week used for statistical analysis. Open circles: NE-BKO mice, black circles: control mice. The data are presented as means ± SEM, with statistically significant differences indicated by ∗*p* ≤ 0.05, ns: not significant [also see Figure S2].

**Figure 2:**
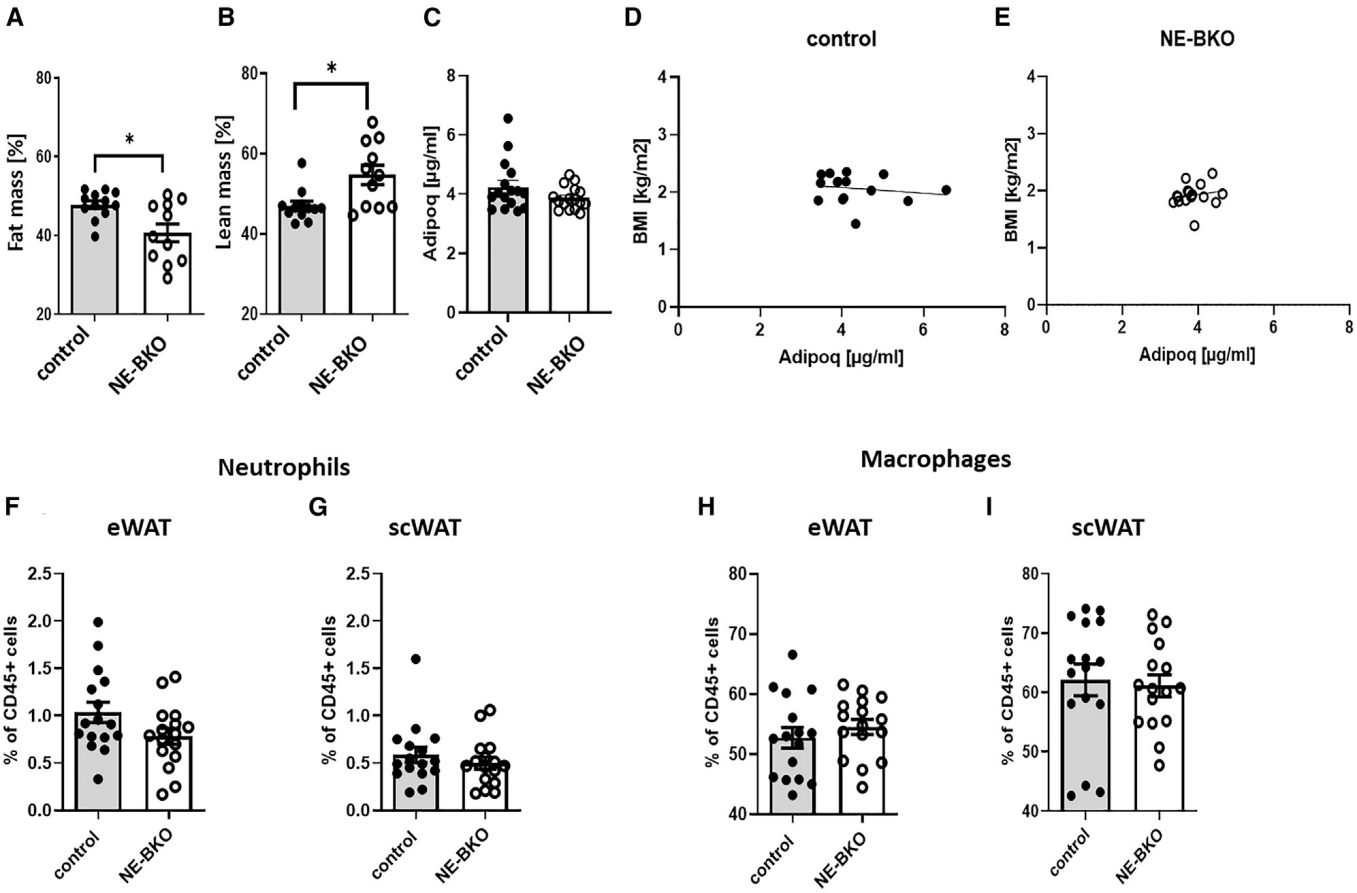
The effect of *Bmal1* deficiency in neutrophils on fat mass, lean mass, adiponectin concentrations, and numbers of pro-inflammatory cells during the first 7 days of HFD In NE-BKO mice, HFD intervention results in reduced body fat mass (t-test, control vs. NE-BKO, t = 2.85, df = 13.85, *p* = 0.013, control *n* = 12, NE-BKO *n* = 11) (A) and increased lean mass (B) compared to control mice (t-test, control vs. NE-BKO, t = 2.88, f = 14.24, *p* = 0.012, control *n* = 12, NE-BKO *n* = 11). However, adiponectin concentrations (control *n* = 16, NE-BKO *n* = 16) (C–E) and leukocyte and macrophage counts in both epididymal and subcutaneous adipose tissue (eWAT and scWAT, respectively, control *n* = 16, NE-BKO *n* = 16) remain comparable between the two groups (F–I). NE-BKO mice are represented by open circles, while control mice are denoted by black circles. The data are presented as means ± SEM, with statistically significant differences indicated by ∗*p* ≤ 0.05 [also see Figure S4].

To address this, we first confirmed that *Mrp8-Cre*-mediated recombination occurs specifically in neutrophils, as evidenced by the distinct band patterns observed (Figure 1A). Importantly, *Bmal1* deletion was exclusive to neutrophils, with no evidence of recombination in other immune cells, such as macrophages, monocytes, and lymphocytes. This confirmation ensured the specificity of the genetic manipulation, allowing us to investigate the role of neutrophil-specific *Bmal1* deficiency in HFD-induced inflammation. Interestingly, while global deficiency of the essential circadian clock gene *Bmal1* significantly affects metabolic processes and overall activity,^12^ restricting *Bmal1* deficiency to neutrophils did not result in marked alterations in locomotor activity, free-running period regulation, food consumption profiles or blood cell counts under normal dietary conditions (Figures S2 and S3). These results suggest that *Bmal1* deficiency in neutrophils does not affect the central circadian clock or contribute to an overt immune phenotype. However, when neutrophil *Bmal1* knockout (NE-BKO) mice were fed HFD, they showed a significant reduction in body weight gain within 7 days compared to control mice (Figure 1B). Notably, this reduction in weight gain occurred although NE-BKO mice consumed the same amount of food as the controls (Figure 1C) and exhibited similar levels of activity (Figures 1D–1F). Moreover, the change in body weight in NE-BKO mice was accompanied by a reduction in body fat (Figure 2A) and an increase in lean mass (Figure 2B) compared to control mice. During this short-term HFD, serum adiponectin levels and the number of neutrophils and macrophages (M1 and M2) in both eWAT and scWAT were similar between control and NE-BKO mice (Figures 2C–2I).

**Figure 3:**
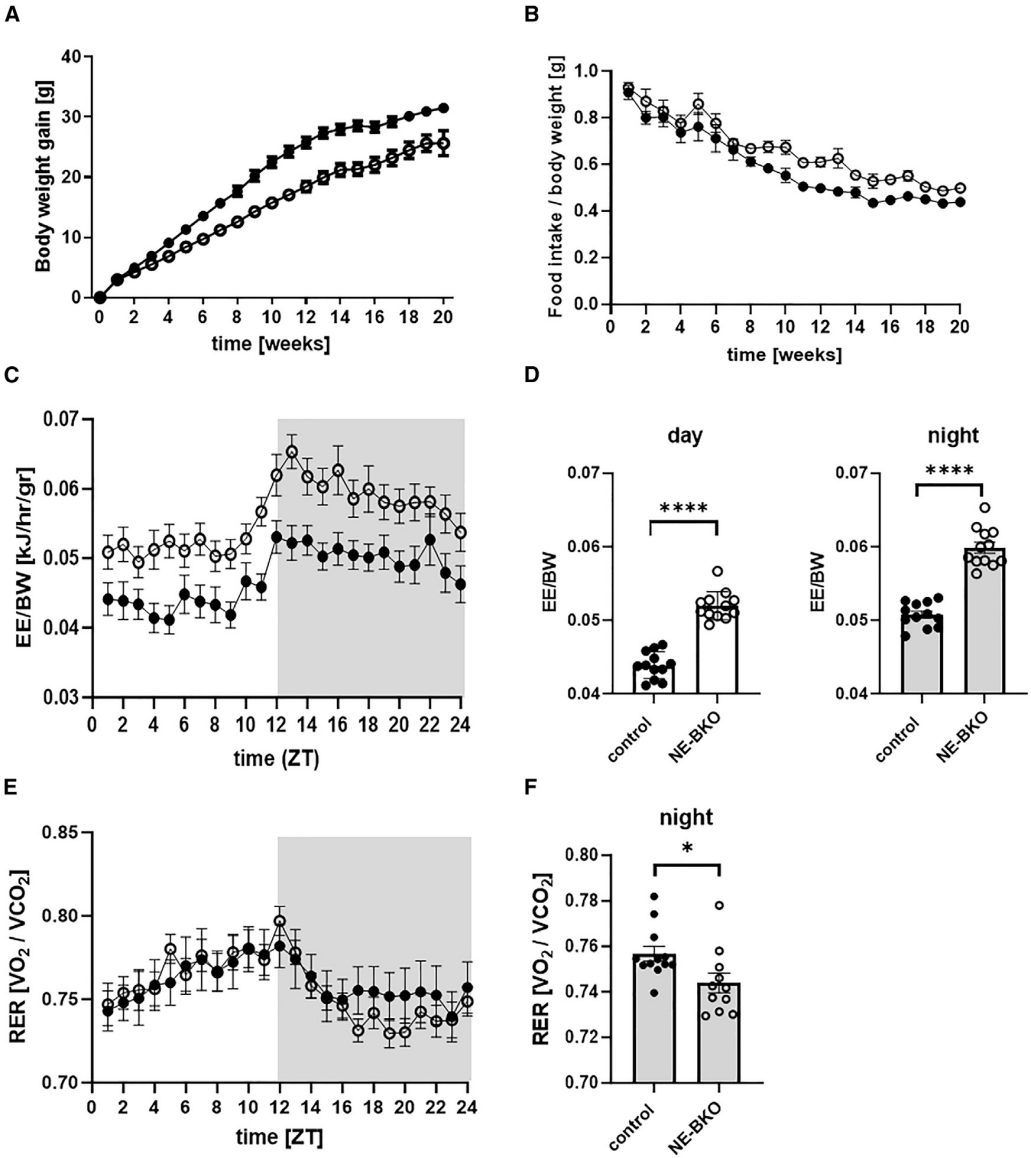
Effect of *Bmal1* deficiency in neutrophils on body mass gain, food intake, and energy expenditure during chronic HFD (A and B) NE-BKO mice exhibit a significantly reduced body weight gain (two-way ANOVA, control vs. NE-BKO, F_(20,160)_ = 15.69, *p* < 0.0001, control *n* = 32, NE-BKO *n* = 32) and increased food intake despite equal activity (counts/min) compared to control mice (two-way ANOVA, control vs. NE-BKO, F_(1,31)_ = 11, *p* = 0.0023, 8th to 16th week, post-hoc *p* < 0.0001, control *n* = 32, NE-BKO *n* = 32). Remarkably, these changes are accompanied by higher energy expenditure on 16^th^ week of HFD during day and night phase in NE-BKO mice compared to control mice (C) (two-way ANOVA, F_(23,36.8)_ = 1.63, *p* < 0.05) (D) t-test, day: t = 15.31, df = 11, *p* < 0.0001, (D) (night: t = 16.37, df = 11, *p* < 0.0001). (E) Overall, both genotypes exhibit similar average total RER, indicating comparable fat utilization for energy acquisition during HFD, with a significant time-dependent effect (two-way ANOVA: NE-BKO vs. control: F_(1,16)_ = 0.05, *p* = 0.83; time effect: F_(7.77, 124.07)_ = 10.07, *p* < 0.0001). (F) However, NE-BKO mice show a marked reduction in RER levels during the night phase compared to control mice (t-test, night: t = 2.40, df = 21, *p* = 0.026). Open circles: NE-BKO mice, black circles: control mice. Gray shaded area depicts the night phase. Data shown are means ± SEM. Significant differences between groups were analyzed by two-way ANOVA, post-hoc Tukey. Statistically significant differences are indicated by ∗∗∗∗*p* ≤ 0.0001 [also see Figure S7].

Remarkably, the smaller body weight gain in NE-BKO mice compared to control mice persisted throughout the 20 weeks of HFD (Figure 3A). Surprisingly, from the 8th to the 16th week of HFD, we observed a significant increase in food intake per body weight (Figure 3B), while body weight gain remained lower compared to the control group (post-hoc *p* < 0.0001). This reduction in body weight gain and increase in food intake per body mass in NE-BKO mice could not be attributed to altered locomotor activity as it mirrored that of control mice (Figure 1D). Furthermore, NE-BKO mice consistently maintained a significantly lower body mass index (BMI) than control mice at both the first and 20th week of HFD exposure (Figure S4). Specifically, the BMI of NE-BKO mice significantly rose from week 1 to week 20 of the HFD, while control mice exhibited no change in BMI (Figure S4).

### Brain and muscle Arnt-like protein-1 deficiency in neutrophils results in increased energy expenditure over 24 h during high-fat diet

Our data suggested that the changes in body mass and food intake observed in NE-BKO mice during HFD were linked to changes in energy expenditure. Energy expenditure was measured over a 24-h on the 16^th^ week of HFD, which was chosen because of the significant differences in body mass gain and food intake between the two groups of animals at this timepoint (Figures 3A and 3B). While hourly EE did not differ between genotypes (Figure S7A), regression analysis showed a significant positive correlation between body weight and EE in both groups (Figures S7B and S7C). To account for body weight effects, EE was normalized, revealing higher EE during the dark (active) phase compared to the light (resting) phase in both genotypes (Figure 3C). Importantly, NE-BKO mice exhibited consistently higher EE throughout the day and night compared to controls (Figure 3D). This increase in EE coincided with changes in respiratory exchange ratio (RER), reflecting fat as the major energy source in both groups. Average RER was similar between control (0.751 ± 0.114) and NE-BKO mice (0.757 ± 0.071), indicating a shared reliance on fat metabolism during HFD. However, NE-BKO mice displayed significantly lower RER during the night phase compared to controls, suggesting differences in nocturnal metabolic adaptation (Figures 3E and 3F). These results suggested that the observed decrease in body mass and increase in food intake was associated with an altered metabolism characterized by increased energy expenditure and reduced RER, particularly during the dark phase, in NE-BKO mice compared to control mice.

### Brain and muscle Arnt-like protein-1 deficiency in neutrophils results in increased UCP1 activity in iBAT after chronic high-fat diet feeding

**Figure 4:**
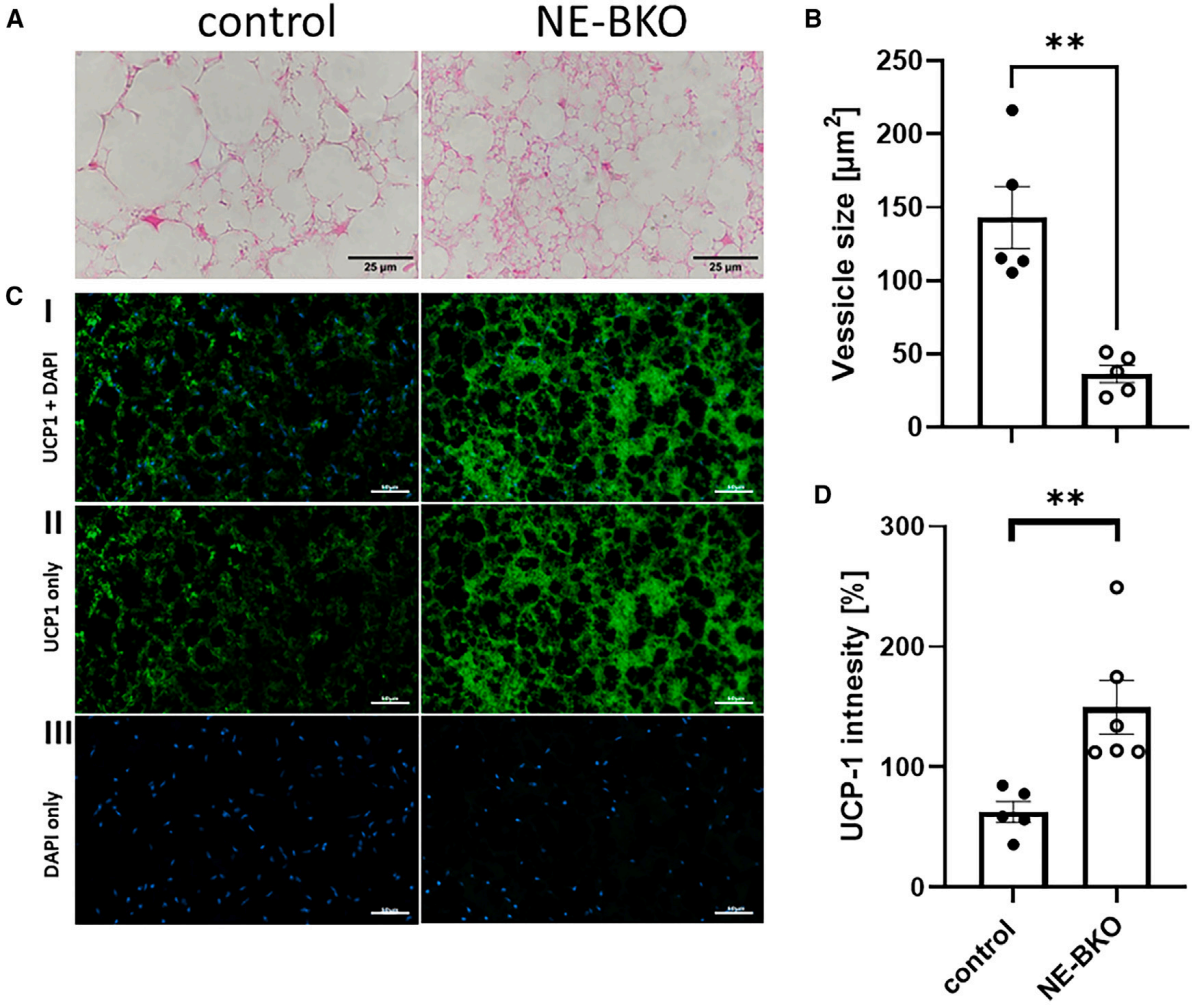
UCP1 expression in iBAT tissue following chronic HFD feeding (A) Representative histological images of H&E-stained iBAT sections from control and NE-BKO mice, captured at 20x magnification, scale bar 25μm. (B) Quantification of vesicle size in iBAT shows a significant increase in NE-BKO mice compared to controls following 20 weeks of chronic HFD (U-test, NE-BKO vs. control: U = 0, *p* = 0.008, control *n* = 5, NE-BKO *n* = 5). (C and D) The larger vesicle size in NE-BKO mice correlates with enhanced UCP1 activity, as indicated by higher fluorescence intensity (U-test, NE-BKO vs. control: U = 0, *p* = 0.004, control *n* = 5, NE-BKO *n* = 6). UCP1 expression was visualized using UCP1-specific antibody conjugated to the Alexa 488 secondary antibody, with nuclei counterstained using DAPI. Panel I shows both UCP1 staining and DAPI staining, panel II shows UCP1 antibody staining alone, and panel III shows DAPI staining only, scale bar 50μm. Data are expressed as means ± SEM. Open circles: NE-BKO mice, black circles: control mice. Orange arrows highlight areas of specific staining and colocalization with DAPI in addition, they also represent an example of this colocalization. Significant differences between groups were assessed using the U-test. ∗∗*p* ≤ 0.01 indicates statistically significant differences.

The shift in RER toward fat oxidation suggests greater fat utilization as an energy source, raising the question of which adipose tissue contributes to this process in NE-BKO mice, leading to reduced body weight gain despite comparable food intake during HFD. To investigate this, we examined the histology of eWAT and iBAT. While vesicle size in eWAT was similar between genotypes, fat content (% fat) was significantly lower in NE-BKO mice (see Figures S6A, S6B, and 2A). In contrast, iBAT in NE-BKO mice contained a higher number of smaller cells compared to controls, indicating enhanced fat mobilization and utilization (Figures 4A and 4B). Immunofluorescence analysis further revealed significantly higher UCP1 expression in NE-BKO mice, as evidenced by increased fluorescence intensity, suggesting elevated thermogenic activity and heat production (Figures 4C and 4D). These findings indicate that increased energy expenditure, driven by enhanced fat oxidation and thermogenesis in iBAT, underlies the reduction in fat mass observed in NE-BKO mice.

### Brain and muscle Arnt-like protein-1 deficiency in neutrophils improves systemic glucose uptake and leads to higher adiponectin levels during the high-fat diet

**Figure 5:**
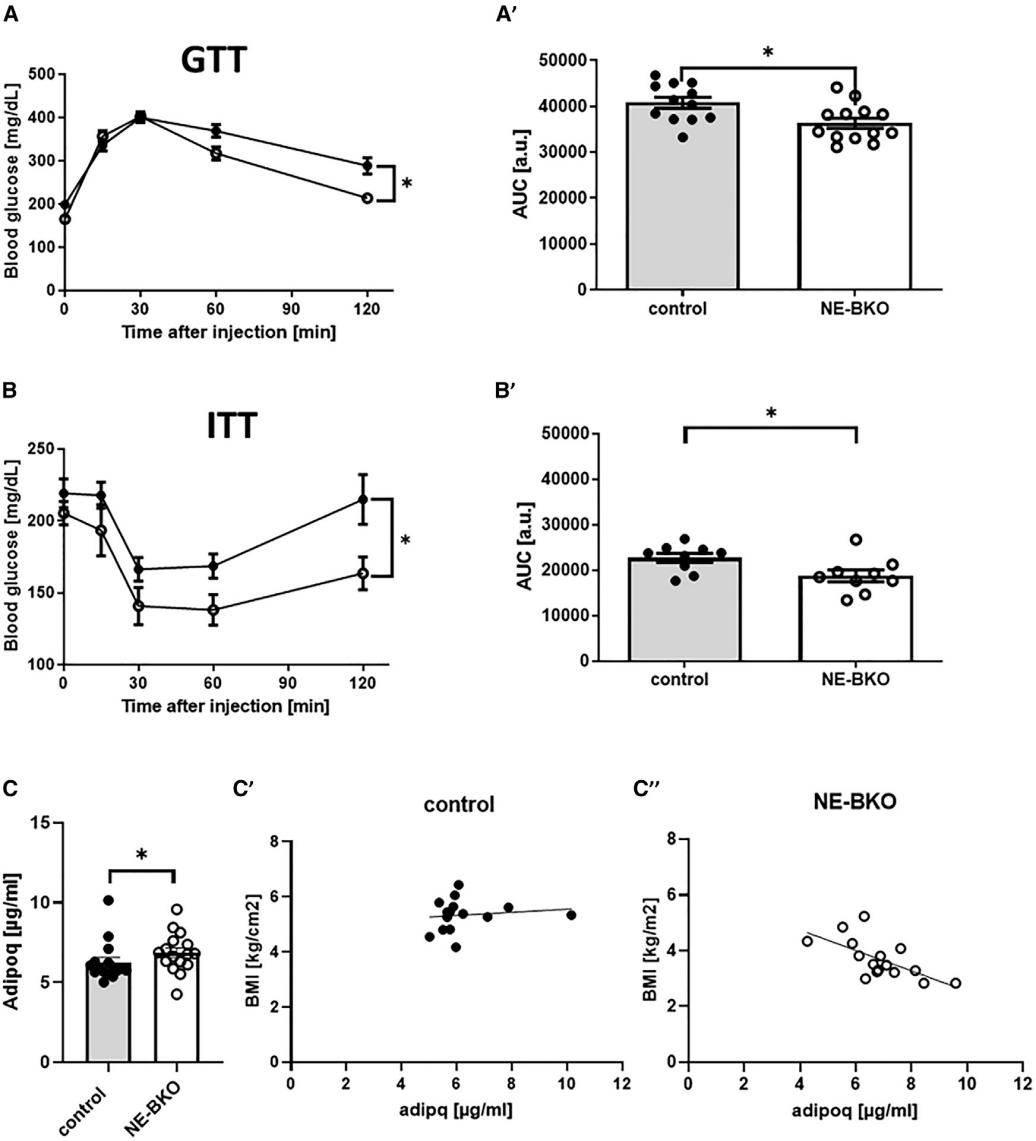
*Bmal1* deficiency and its effects on glucose tolerance, insulin sensitivity, and adiponectin levels during chronic HFD *Bmal1* deficiency in neutrophils during chronic HFD results in increased glucose uptake (A-A′) and increased insulin sensitivity (B-B') (two-way ANOVA, GTT: F_(4,41)_ = 6.125, *p* < 0.001, 120 min post-hoc *p* < 0.05, control *n* = 15, NE-BKO *n* = 13; ITT: F_(4,48)_ = 22.22, *p* < 0.0001, 120 min post hoc *p* < 0.05, control *n* = 9, NE-BKO *n* = 9). In addition, adiponectin levels are significantly elevated (U-test, U = 71, *p* = 0.031, control *n* = 16, NE-BKO *n* = 16) and show a negative correlation with BMI in NE-BKO mice compared to control mice (C-C’’) (NE-BKO and BMI: Spearman, R = −0.712, *p* = 0.003, control *n* = 16, NE-BKO *n* = 16). NE-BKO: *Bmal1* deficiency in neutrophils, BMI: body mass index, GTT: glucose tolerance test, ITT: insulin tolerance test, AUC area under the curve. Open circles: NE-BKO mice, black circles: control mice. Data shown are means ± SEM. Significant differences between groups were analyzed by two-way ANOVA, post-hoc Tukey ∗*p* ≤ 0.05; Spearman correlation analysis was used to assess the association between BMI and adiponectin concentration [also see Figure S5].

The enhanced fat mobilization and thermogenic activity observed in iBAT of NE-BKO mice likely play a role in their altered systemic metabolism. These processes not only reduce fat mass but also influence nutrient handling and energy homeostasis. The observed increase in energy expenditure and reduced adiposity prompted us to investigate whether these changes affect glucose metabolism and insulin sensitivity during HFD. Remarkably, NE-BKO mice exhibited increased insulin sensitivity and glucose uptake compared to control mice by the 20th week of HFD (Figures 5A, 5A′, 5B, and 5B′). This finding corresponded with the observed negative correlation of BMI with adiponectin concentration in the last week of HFD exposure in NE-BKO mice (Figures 5C and 5C′). Conversely, control mice, which had lower serum adiponectin levels compared to NE-BKO, showed no correlation between BMI and adiponectin levels neither in the first week nor in the 20th week of HFD. (Figures 2D and 5D–5D″). This inverse correlation aligns with the established understanding that lower adiposity tends to be associated with higher adiponectin levels.^37^

### Lack of brain and muscle Arnt-like protein-1 in neutrophils leads to changes in both M1-and M2 macrophage composition in epididymal adipose tissue and subcutaneous adipose tissue

**Figure 6:**
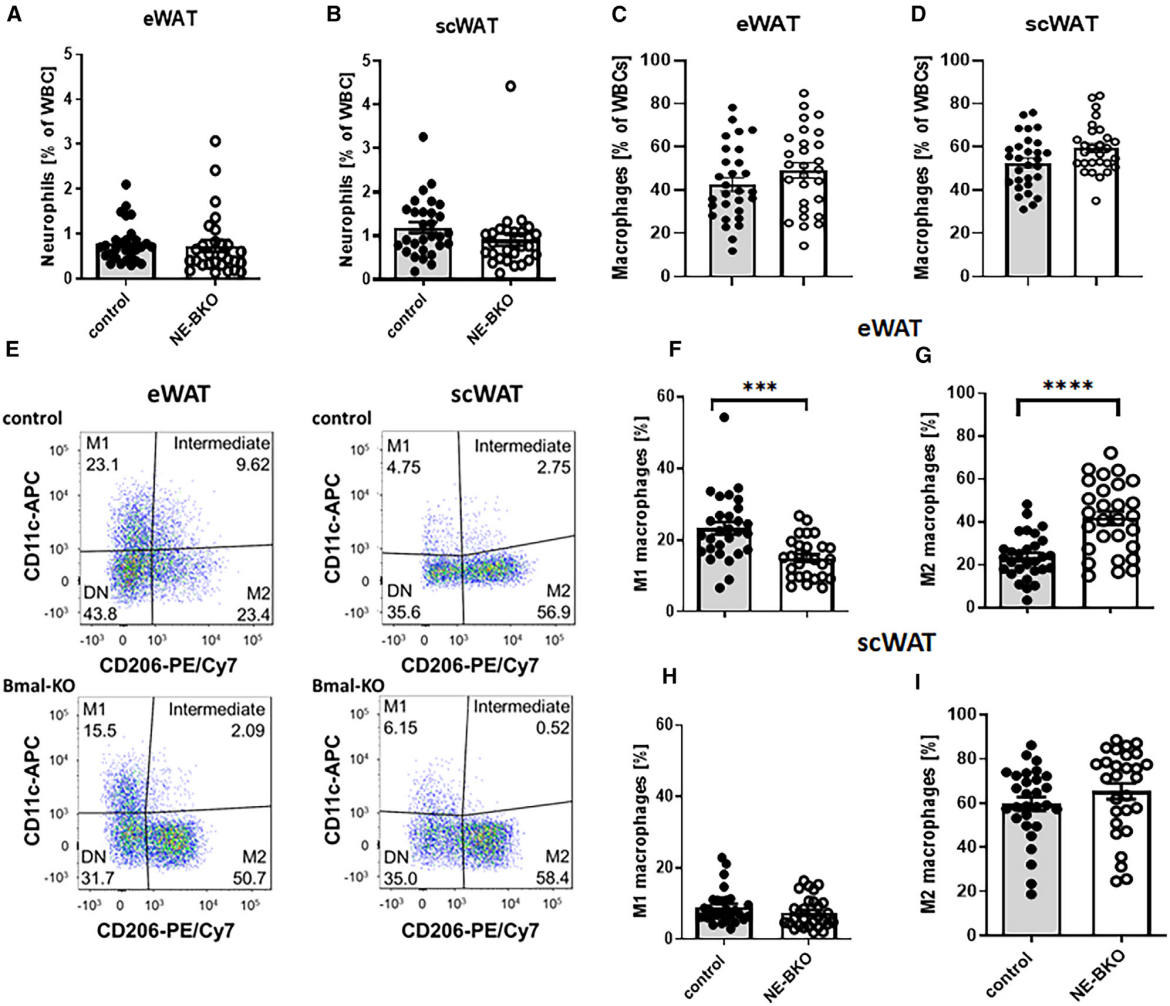
Effect of *Bmal1* deficiency in neutrophils on the number of macrophages and their polarisation in eWAT and scWAT after long-term HFD feeding Number of neutrophils and macrophages as percentage in eWAT (t-test, neutrophils: t = 0.29, df = 55, *p* = 0.699; macrophages, t = 1.36, df = 55, *p* = 0.180, control *n* = 29, NE-BKO *n* = 28) and in scWAT on the 20^th^ day of HFD (t-test, neutrophils: t = 1.45, df = 55, *p* = 0.154; macrophages: t = 2.14, df = 55, *p* = 0.603, control *n* = 29, NE-BKO *n* = 28) (A and B). Representative images from macrophage analysis and fat pad weight of eWAT or scWAT (E). Number of M1-and M2-like macrophages as percentage in eWAT (t-test, M1 control vs*.* NE-BKO: t = 4.12, df = 46.28, *p* = 0.0002, M2 control vs*.* NE-BKO, t = 4.90, df = 45.55, *p* < 0.0001, control *n* = 29, NE-BKO *n* = 28) (C, F, and G). Number of M1-like and M2-like macrophages as a percentage in scWAT of NE-BKO mice did not differ significantly from the control mice (t-test, M1: t = 1.31, df = 54.69, *p* = 0.196, M2: t = 1.19, df = 52.92, *p* = 0.239, control *n* = 29, NE-BKO *n* = 28) (D, H, and I). Data were averaged across the whole 24-h cycle (M1: M1-like macrophages, M2: M2-like macrophages). Open circles: NE-BKO mice, black circles: control mice. Data are presented as means ± SEM. Significant differences between both groups NE-BKO and control were analyzed using paired t-test ∗∗∗*p* ≤ 0.001, ∗∗∗∗*p* ≤ 0.0001. [also see Figure S1].

**Figure 7:**
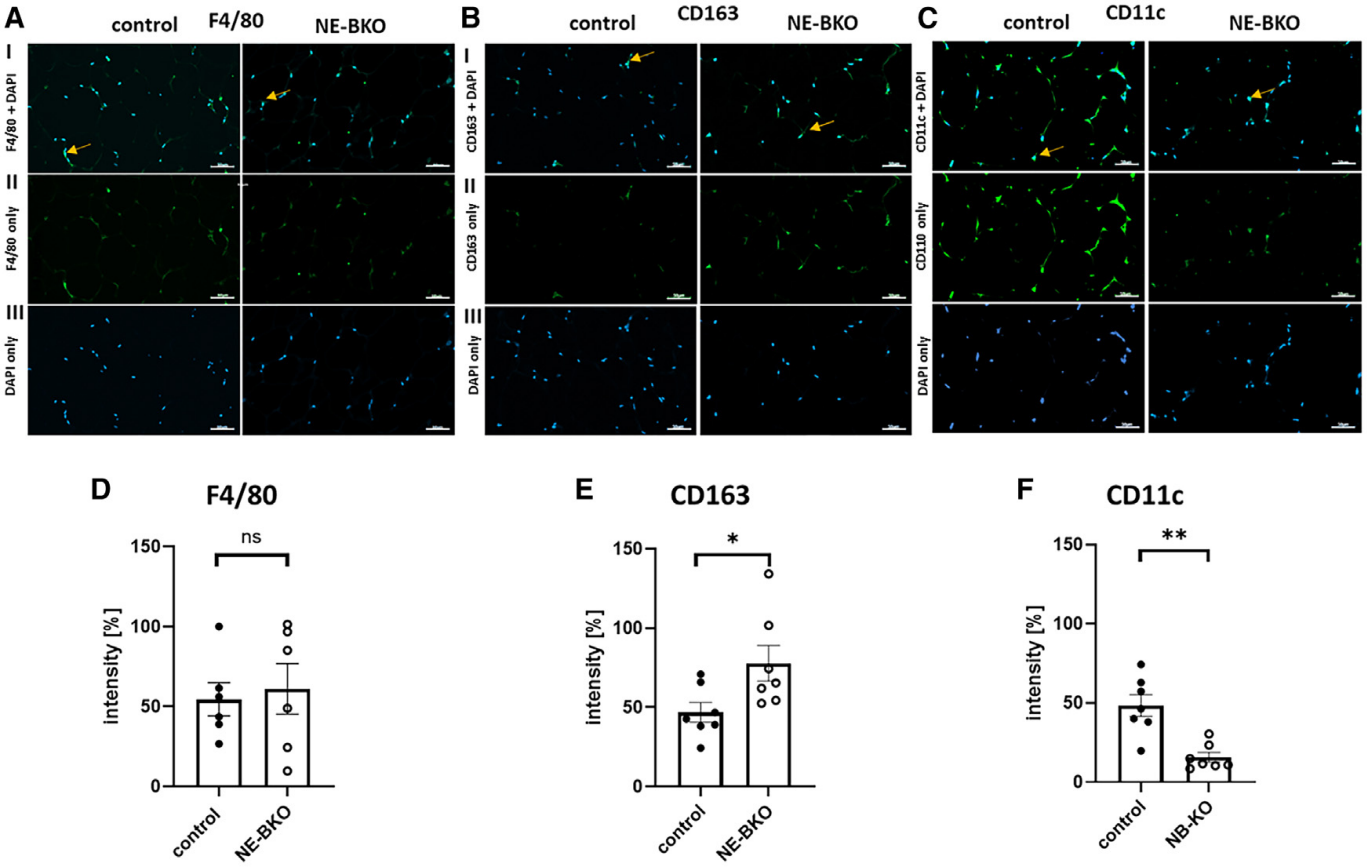
Effect of *Bmal1* deficiency in neutrophils on macrophage infiltration into eWAT (A–C) Representative images of eWAT tissue sections stained for F4/80 (macrophage marker), CD163 (marker for macrophage type 2, M2), and CD11c (marker for macrophage type 1, M1); captured at 20x magnification, scale bar 50μm. The number of positively stained cells correlates with the intensity of the primary antibody conjugated to Alexa 488 secondary antibody. (D) Quantification of F4/80 staining shows no significant difference in macrophage infiltration between NE-BKO and control mice (U-test, NE-BKO vs. control: U = 17, *p* = 0.937, control *n* = 6, NE-BKO *n* = 6). (E and F) However, when dividing the macrophage populations into M2 and M1 subsets, NE-BKO mice exhibit significantly increased M2 infiltration and decreased M1 infiltration compared to control mice (U-test, NE-BKO vs. control, CD163: U = 8, *p* = 0.038; CD11c: U = 2, *p* = 0.0023, control *n* = 6, NE-BKO *n* = 6).Panel I shows combined staining for F4/80, CD163, or CD11c, along with DAPI staining. Panel II displays staining for F4/80, CD163, or CD11c alone, and panel III shows DAPI staining only. Open circles: NE-BKO mice, black circles: control mice. Data are expressed as means ± SEM. Statistical significance between groups was assessed using the U-test. ∗*p* ≤ 0.05, ∗∗*p* ≤ 0.01 indicates statistically significant differences [also see Figure S6].

The improved glucose uptake, increased adiponectin levels, and lower body mass gain observed in NE-BKO mice compared to control mice during HFD suggested a potential reduction in pro-inflammatory cell responses in metabolically active adipose tissues, such as eWAT and scWAT. To investigate this further, we assessed the proportions of tissue-resident pro-inflammatory cells, i.e., neutrophils and macrophages, in these depots. Interestingly, although the percentage of neutrophils and macrophages was comparable between NE-BKO and control mice (Figures 6A–6D), NE-BKO mice exhibited a reduced proportion of pro-inflammatory M1 and an increased proportion of anti-inflammatory M2 macrophages in eWAT, leading to a higher M2/M1 ratio compared to control mice (Figures 6F and 6G). Conversely, in scWAT, there were no significant changes in the percentage of macrophages or M1 and M2 ratios between the two groups (Figures 6H and 6I). To confirm these findings, we performed immunofluorescence analysis on eWAT tissue. The fluorescence intensity of the general macrophage marker F4/80 was similar between genotypes (Figures 7A and 7D). However, NE-BKO mice displayed increased intensity of the M2 macrophage marker (CD163)^38^ compared to controls (Figures 7B and 7E). Conversely, control mice showed higher expression of the pro-inflammatory M1 marker (CD11c)^39^ in eWAT than NE-BKO mice (Figures 7C and 7F).

These results from both flow cytometry and immunofluorescence analyses indicate that *Bmal1* deficiency in neutrophils selectively promotes anti-inflammatory M2 macrophage infiltration in eWAT, while scWAT remains unaffected. This depot-specific response suggests that neutrophil-specific *Bmal1* deficiency modulates the inflammatory profile of eWAT, potentially mitigating the inflammatory effects of chronic HFD.

### Neutrophils lacking brain and muscle Arnt-like protein-1 show reduced expression of pro-inflammatory but elevated anti-inflammatory genes commonly associated with M2 macrophages in epididymal adipose tissue

**Figure 8:**
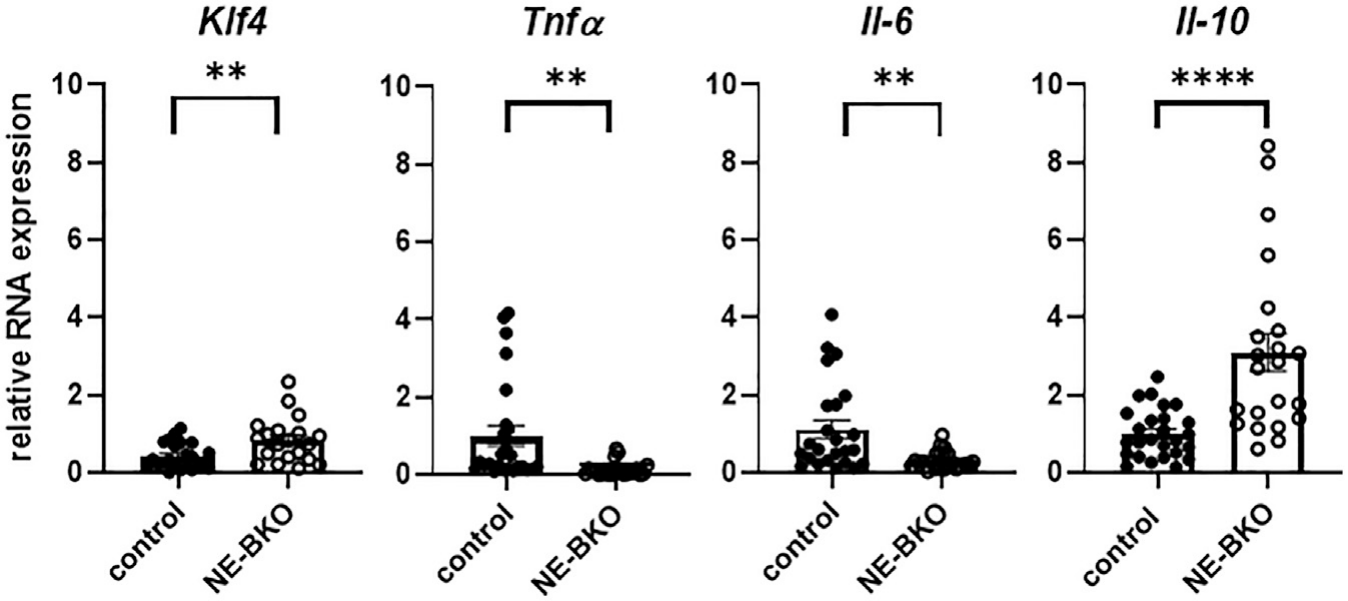
Effect of *Bmal1* deficiency in neutrophils on gene expression in eWAT, encoding pro- and anti-inflammatory cytokines and *Klf4* after 20 weeks of HFD feeding In NE-BKO mice, the total relative mRNA expression of *Klf4* is significantly increased (t-test, t = 2.83, df = 29.43, *p* = 0.0084, control *n* = 26, NE-BKO *n* = 23). Conversely, the expression of genes encoding the pro-inflammatory factors *Tnfα* and *Il-6* is significantly reduced in NE-BKO mice compared to control mice (t-test, *Tnfα*: t = 3.22, df = 25.12, *p* = 0.0035; *Il-6*: t = 3.324, df = 24.96, *p* = 0.0027, control *n* = 26, NE-BKO *n* = 23). In contrast to pro-inflammatory factors, the expression of the anti-inflammatory cytokine *Il-10* is significantly elevated in NE-BKO compared to control mice (t-test, t = 4.308, df = 24.96, *p* = 0.0002, control *n* = 26, NE-BKO *n* = 23). Filled circles: control mice, open circles: NE-BKO, data are presented as means ± SEM. Significant differences in total RNA expression between both groups (NE-BKO and control) were analyzed using unpaired t-test, ∗∗*p* ≤ 0.01, ∗∗∗*p* ≤ 0.001, ∗∗∗∗*p* ≤ 0.0001 [also see Figure S8].

We further investigated whether the increased M2/M1 ratio observed in the eWAT of NE-BKO mice corresponded to the increased expression of *Krueppel-like factor 4* (*Klf4*), a marker of M2 cell polarisation. After 20 weeks of HFD, we monitored *Klf4* expression in eWAT over a 24-h period. Notably, NE-BKO mice exhibited a significantly higher *Klf4* expression compared to control mice (Figure 8A: *Klf4*).

In addition, a higher proportion of M2 macrophages in NE-BKO mice was associated with decreased mRNA levels of pro-inflammatory mediators, *Tnfα* and *Il-6*, which are typically produced by M1 macrophages.^40^ Interestingly, the expression patterns of *Klf4* were inversely related, with *Tnfα* levels lower in NE-BKO mice when *Klf4* expression was elevated. In contrast, the control group exhibited significantly higher total levels of pro-inflammatory factors compared to NE-BKO mice (Figure 8A: *Tnfα*, *Il-6*). Additionally, the gene encoding *Il-10*, a cytokine with anti-inflammatory properties produced by several immune cells, including M2,^41^^,^^42^ showed significantly elevated total expression in NE-BKO mice compared to controls (Figure 8: *Il-10*). However, under chow diet conditions, the expression of *Klf4*, *Tnfα*, *Il-6*, and *Il-10* was comparable between the two genotypes (see Figure S8), suggesting that the anti-inflammatory profile observed in NE-BKO mice is a result of chronic HFD exposure. These findings indicate that the absence of *Bmal1* in neutrophils alters the inflammatory response to HFD, promoting an anti-inflammatory environment in NE-BKO mice.

### The absence of brain and muscle Arnt-like protein-1 in neutrophils correlates with the decreased gene expression of chemokine ligand-2 receptor 2 and its ligand chemokine ligand-2, and increased expression of *Cxcr4 in* epididymal adipose tissue during chronic high-fat diet

Building upon the distinct expression profiles of pro-inflammatory genes observed in NE-BKO and control mice in eWAT, we hypothesized that genes involved in facilitating neutrophil granulocyte infiltration into the tissue might exhibit differential expression patterns, influencing the immune response to infectious agents.^43^ To explore this, we analyzed the gene expression of *Cxcr2*, its ligand *Cxcl2*, and *Cxcr4* in eWAT obtained from both NE-BKO and control mice under HFD and chow conditions.

**Figure 9:**
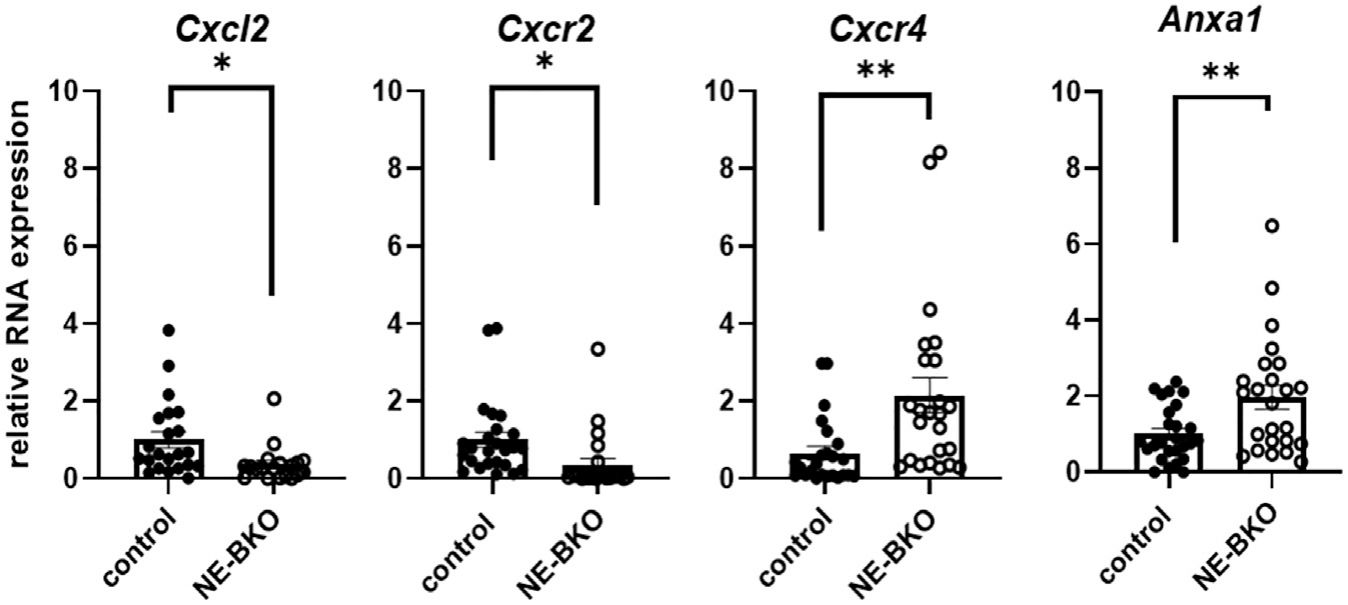
Effect of *Bmal1* deficiency in neutrophils on gene expression in eWAT, encoding proteins that regulate neutrophil migration into tissues after prolonged exposure to HFD While gene expression for the chemokine receptor CXCR2 and the chemokine ligand Cxcl2 is significantly reduced in NE-BKO mice compared to control mice (t-test, *Cxcl2* NE-BKO vs. control: t = 2.745, df = 31.17, *p* = 0.0100; *Cxcr2* NE-BKO vs. control: t = 2.544, df = 44.79, *p* = 0.0145, control *n* = 26, NE-BKO *n* = 23), the expression of *Cxcr4* is significantly elevated in NE-BKO mice (t-test, NE-BKO vs. control: t = 3.060, df = 29.80, *p* = 0.0047, control *n* = 26, NE-BKO *n* = 23). Also, the gene expression of *Anxa1* is elevated compared to controls (t-test, NE-BKO vs. control: t = 2.837, df = 32.26, *p* = 0.0078, control *n* = 26, NE-BKO *n* = 23). Filled circles: control mice, open circles: NE-BKO, data are presented as means ± SEM. Significant differences in total mRNA expression between both groups (NE-BKO and control) were analyzed using unpaired t-test, ∗∗*p* ≤ 0.01, ∗∗∗*p* ≤ 0.001, ∗∗∗∗*p* ≤ 0.0001 [also see Figure S8].

Our results showed a decrease in the total gene expression of *Cxcl2* and the gene encoding *CXCR2* in NE-BKO mice (Figure 9: *Cxcl2*, *Cxcr2*) compared to the control group. Conversely, the control group showed a general increase in both gene expressions (Figure 9: *Cxcl2*, *Cxcr2*). Furthermore, the gene encoding CXCR4, which counteracts the activity of CXCR2 and thereby prevents neutrophil migration into tissues,^18^ showed significantly higher RNA expression levels in NE-BKO mice compared to control mice. We further examined the expression of *Anxa1*, a gene encoding Annexin A1, which inhibits neutrophil migration and, downregulates of pro-inflammatory mediators.^27^^,^^44^ In HFD-fed NE-BKO mice, *Anxa1* expression was significantly higher than in controls (Figure 9: *Anxa1*), suggesting a mechanism that reduces neutrophil infiltration in eWAT.

In contrast, under chow diet conditions, the expression of *Cxcl2* and *Cxcr2* was generally lower in NE-BKO mice compared to controls but did not reach statistical significance (see Figure S8: *Cxcl2*, *Cxcr2*). Notably, *Cxcr4* expression was significantly lower in NE-BKO mice under chow conditions compared to controls, a reversal of the pattern seen under HFD (see Figure S8: *Cxcr4*; Figure 9: *Cxcr4*). Similarly, *Anxa1* expression was comparable between genotypes on chow but was significantly higher in HFD-fed NE-BKO mice compared to control mice (see Figure S8: *Anxa1*; Figure 9: *Anxa1*). These findings suggest that *Bmal1* deficiency may affect neutrophil migration into eWAT based on dietary context. Under HFD conditions, the upregulation of *Cxcr4* and *Anxa1*, combined with the downregulation of *Cxcl2* and *Cxcr2*, points to altered neutrophil infiltration and a shift toward an anti-inflammatory profile in NE-BKO mice. Additionally, considering that aging neutrophils tend to decrease *Cxcr2* while increasing *Cxcr4* expression,^45^^,^^46^^,^^47^ the findings suggest that *Bmal1* deficiency in neutrophils may accelerates their aging process. This acceleration is characterized by lower levels of *Cxcl2* and *Cxcr2*, together with higher levels of *Cxcr4* gene expression compared to control mice.

## Discussion

HFD promotes obesity and activates pro-inflammatory pathways in metabolic tissues, disrupting circadian clocks in peripheral tissues and shedding light on the physiological mechanisms of obesity.^30^^,^^48^^,^^49^^,^^50^^,^^51^^,^^52^ Tissue specific mutations or deletions of clock genes, such as *Bmal1* in adipose tissue, highlight the importance of adipocyte clocks in energy homeostasis.^11^ However, the impact of global *Bmal1* deficiency on body weight and metabolism under HFD remains controversial, with studies showing both exacerbation and protection from metabolic dysfunction.^13^^,^^14^^,^^53^^,^^54^

Unlike the pro-inflammatory effects typically associated with *Bmal1* deficiency in macrophages and monocytes, which have been shown to increase *Cxcl2* and *Cxcr2* expression, elevate M1 macrophage levels, and enhance neutrophil recruitment under inflammatory conditions such as HFD exposure,^55^^,^^56^ our study demonstrates that neutrophil-specific *Bmal1* deficiency produces a distinct anti-inflammatory phenotype under chronic HFD. In NE-BKO mice, this phenotype is characterized by the reduced expression of *Cxcl2* and *Cxcr2*, alongside increased *Cxcr4* expression, which is associated with pro-inflammatory cell recruitment and promotes higher levels of anti-inflammatory M2 macrophages following chronic HFD exposure. Supporting these findings, NE-BKO mice under HFD conditions exhibit decreased expression of pro-inflammatory genes (*Tnfα*, *Il-6*) and increase in anti-inflammatory gene *Il-10* in metabolically active eWAT, slower weight gain, increased energy expenditure, and enhanced UCP1 expression in iBAT. This divergence in the effects of *Bmal1* deficiency between neutrophils and macrophages highlights that the observed phenotype specifically arises from *Bmal1* deletion in neutrophils, rather than from potential leakage of *Mpr8-Cre* recombinase into monocytes or macrophages. This conclusion is further supported by genotyping results. Collectively, these findings emphasize how the altered chemokine signaling, due to neutrophil-specific *Bmal1* deficiency, drives the distinct inflammatory and metabolic adaptations observed in NE-BKO mice under HFD conditions.

Specifically, the observed reduction in inflammation and lack of significant body weight gain in NE-BKO mice in response to HFD is likely due to altered cell-intrinsic signaling involving the chemokines Cxcl2 and CXCR2, which are regulated by BMAL1.^18^^,^^57^ These chemokines are crucial for aging and the rhythmic trafficking of neutrophils across tissues under both baseline and inflammatory conditions.^58^ Consistent with findings of Adrover and colleagues,^5^^,^^18^ we observed that *Bmal1* deficiency in neutrophils leads to the reduced expression of *Cxcl2* and *Cxcr2* in NE-BKO mice, a pattern that we observed under both chow and HFD conditions. Additionally, *Cxcr4* expression was significantly upregulated in NE-BKO mice on HFD compared to control mice and was notably higher than in NE-BKO mice on chow. According to Dalli et al. 2012,^45^ the downregulation of CXCR2 is often accompanied by an upregulation of CXCR4 on the surface of neutrophils. This shift in chemokine expression is known to extend neutrophil lifespan and aligns with a senescent neutrophil phenotype.^45^^,^^59^^,^^60^ Hence, the observed elevated *Cxcr4* expression suggests that neutrophils in NE-BKO mice, particularly under HFD conditions, may exhibit increased features of senescence or aging, contributing to the altered inflammatory response. It has already been suggested by Adrover et al. 2020^5^ that aging neutrophils show a progressive loss of granule proteins involved in inflammation and altered NET formation. This reduced functionality may protect against excessive leukocyte activity, potentially explaining the lack of inflammation in the eWAT of NE-BKO mice, as evidenced by lower inflammatory gene expression. Supporting this, NE-BKO mice show increased expression of anti-inflammatory genes such as *Il-10* and decreased expression of pro-inflammatory factors such as *Tnfα* and *Il-6* in eWAT. Surprisingly, however, both genotypes showed similar levels of neutrophil infiltration in eWAT and scWAT. While on first glance, this finding might appear contradictory, it is important to note that our current results cannot rule out differences in neutrophil behavior, as further investigations — such as tracking neutrophil dynamics — would be required to clarify this point. In contrast, the observed reduction in *Cxcr4* levels in chow-fed control mice likely influenced neutrophil mobilization dynamics from the bone marrow. CXCR4 is well known for its role in retaining neutrophils within the bone marrow, and its decreased expression can result in less effective retention and premature release of neutrophils into circulation.^48^^,^^61^^,^^62^ Under HFD conditions, this may have led in control mice to an increased proportion of immature neutrophils in the bloodstream, which subsequently infiltrated tissues. The infiltration rate of neutrophils is not rhythmic in both control and NE-BKO (see Figure S5), however, this difference in neutrophil aging states could significantly influence the inflammatory environment in **eWAT.** Elevated **CXCR4** levels, as shown by Yao et al. 2014,^63^ are known to limit pro-inflammatory cell recruitment into adipose tissue, potentially reducing the inflammation in eWAT observed in NE-BKO mice in our study. To confirm these differences in neutrophil aging, further studies are required to identify hallmarks of aged neutrophils, such as **hypersegmentation**, changes in cell size, and high expression of **CD62L** and **CXCR4** on the cell surface.^48^^,^^59^^,^^62^

The anti-inflammatory state in **NE-BKO mice**, as observed in our study, is further evidenced in addition to the **low expression of pro-inflammatory factors** such as ***Tnfα*** and ***Il-6***, by changes in macrophage polarization, which together establish an anti-inflammatory environment in **eWAT**. Specifically, **NE-BKO mice** exhibit a higher prevalence of **M2 macrophages** and increased expression of ***Anxa1*** and ***Klf4***, both of which promote M2 polarization and suppress inflammation.^45^^,^^64^^,^^65^^,^^66^^,^^67^ **ANXA1**, predominantly produced by neutrophils,^55^^,^^68^^,^^69^^,^^70^^,^^71^^,^^72^ influences lipid metabolism via the **11β-HSD1 enzyme**,^44^^,^^73^^,^^74^^,^^75^ supporting the observation that **NE-BKO mice** show enhanced fat metabolism. Meanwhile, **KLF4** contributes to decreased M1 macrophage expression,^67^ further contributing to the anti-inflammatory profile of NE-BKO mice. This is further supported by the difference in adiponectin levels between the two genotypes under HFD, where free fatty acids (FFAs) in control mice may downregulate KLF4 and trigger inflammation, as shown in previous studies.^76^^,^^77^ In contrast, **NE-BKO mice** appear to utilize fat more efficiently as indicated by their RER and increased energy expenditure, which helps reduce inflammation even under HFD condition. These findings suggest that in NE-BKO mice, fat, including triglycerides that decompose into FFAs, is utilized by an increased metabolism, mitigating inflammation. This anti-inflammatory state in NE-BKO mice not only supports efficient fat metabolism but also highlights their resilience to diet-induced metabolic stress. These findings provide a foundation for understanding how HFD uniquely alters metabolic and inflammatory profiles in NE-BKO mice, distinguishing them from their chow-fed counterparts.

Of note, NE-BKO mice fed normal chow exhibit no metabolic phenotype or signs of inflammation, despite the absence of rhythmic neutrophil trafficking observed in studies by Adrover et al. 2019.^18^

Additionally, in chow-fed NE-BKO mice, *Cxcl2* and *Cxcr2* expression patterns closely resemble the findings reported by Adrover et al. 2019.^18^ This consistency suggests that the chemokine changes observed in the eWAT of NE-BKO mice are driven by *Bmal1* loss in neutrophils, rather than being influenced by other immune cell populations. Although *Bmal1* deficiency in neutrophils has been linked to enhanced toxicity under lipopolysaccharide (LPS) challenge,^5^ these effects do not disrupt metabolic stability under baseline dietary conditions in our study. In contrast, HFD induces significant metabolic changes in NE-BKO mice, including increased energy expenditure, reduced RER, and elevated *Cxcr4* expression in eWAT. These findings align with research suggesting that CXCR4 signaling enhances energy expenditure and reduces adipose tissue inflammation by limiting inflammatory cell recruitment.^67^ Correspondingly, NE-BKO mice display reduced inflammatory markers in eWAT, higher M2 macrophage levels, slower weight gain, and decreased susceptibility to diet-induced obesity.

Additionally, the increased *Cxcl2* expression in WAT of control mice under HFD conditions aligns with studies showing that HFD induces the overproduction of Cxcl2,^78^^,^^79^^,^^80^^,^^81^ associated with a pro-inflammatory response, including elevated M1 macrophages^82^^,^^83^^,^^84^ and increased *Il-6* and *Tnfα* levels^81^ — patterns confirmed by our study. Intriguingly, NE-BKO mice on HFD also exhibit elevated UCP1 expression in iBAT, indicating enhanced fat mobilization and thermogenesis as an adaptive mechanism to counteract metabolic stress. This increase in UCP1 expression likely underlies the reduced fat mass and slowed weight gain observed in NE-BKO mice. The link between elevated *Cxcr4* expression in eWAT and increased UCP1 expression in iBAT, as suggested by Yao et al. (2014),^63^ warrants further investigation. Together, these findings highlight the role of *Bmal1* in regulating chemokine signaling and metabolic adaptation under dietary stress, promoting resilience against obesity and inflammation in NE-BKO mice. However, the potential risk of increased susceptibility to infection due to senescent neutrophils remains a consideration.

In conclusion, our study demonstrates that *Bmal1* deficiency in neutrophils significantly alters the inflammation in eWAT and lipid accumulation in iBAT, resulting in increased UCP1 expression in iBAT under chronic HFD conditions. These effects also lead to the reduced infiltration of pro-inflammatory macrophages into metabolically active eWAT that may be likely due to altered Cxcl2-CXCR2 signaling, highlighting the critical role of *Bmal1* and the circadian system in maintaining metabolic and inflammatory balance under different nutritional conditions. Building on these findings, we show that specific *Bmal1* deficiency in neutrophils reduces obesity and insulin resistance, aligning with findings by Jouffe et al. 2022^13^ and Zhan et al. 2024.^14^ While our approach indicates a potential cell-specific role of ***Bmal1*** in these metabolic changes, it does not preclude contributions from other cell types, emphasizing the need for further investigation to delineate the broader implications of ***Bmal1*** in metabolic regulation.

### Limitations of the study

Our study demonstrates that neutrophil-specific *Bmal1* deficiency produces a distinct anti-inflammatory phenotype under chronic HFD conditions, associated with enhanced energy expenditure, increased UCP1 expression in iBAT, and altered chemokine gene expression. However, the mechanisms linking these changes to the observed metabolic alterations and reduced body weight gain in NE-BKO mice compared to controls remain unclear and warrant further investigation.

A key question is whether the altered chemokine expression in NE-BKO eWAT originates solely from neutrophils or is influenced by other cell types. Assessing chemokine expression directly in neutrophils would provide valuable insights and address this uncertainty. Furthermore, we observed elevated M2/M1 macrophage ratios in NE-BKO mice during chronic HFD, as demonstrated by FACS and immunofluorescence analyses. While these methods yielded consistent outcomes, a comprehensive evaluation of the total number of infiltrated M1 and M2 macrophages, coupled with an assessment of eWAT weight, could offer a more robust understanding of how *Bmal1* deficiency impacts macrophage infiltration. Notably, potential changes in eWAT weight may also reflect the reduced body weight gain seen in NE-BKO mice.

Finally, the potential role of neutrophil senescence in NE-BKO mice remains unexplored. Following this process through the analysis of Cxcl12-CXCR4 interactions, alongside markers such as CD62L and ROS levels in eWAT and other metabolically active tissues such as iBAT, could provide more direct evidence for altered neutrophil aging and its metabolic implications.

It is important to note that our study exclusively used male mice, and therefore the findings may not be directly applicable to females. Sex differences in immune responses and metabolic regulation have been well-documented, and future studies should investigate whether the observed effects of neutrophil-specific *Bmal1* deficiency under chronic HFD conditions are consistent between males and females.

## Resource availability

### Lead contact

Further information and requests for resources and kits should be directed to the main contact person Dr. Violetta Pilorz (violetta.pilorz@uni-luebeck.de), who will deal with them.

### Materials availability

This study did not generate new unique reagents. There are restrictions to the availability of the NE-BKO mouse line due to the mouse line having been generated exclusively for the purpose of the study. However, the mouse line can be easily recreated as it has been generated by cross breeding commercially available lines.

### Data and code availability

• Data reported in this article will be shared by the lead contact upon request.
• This article does not report original code.
• Any additional information required to reanalyse the data reported in this article is available from the lead contact upon request.

## Acknowledgments

This work was supported by a grant from the 10.13039/501100001659German Research Foundation, German (OS353-10/1) to HO. We thank our animal caretakers, Sven Schroeder and Kerstin Lünsmann, for their excellent support. We are also grateful to Drs. Geisler and Vollbrandt from the Cell Analysis Core Facility at the University of Lübeck for their invaluable assistance with the use of their equipment. We thank Dr. M. Hirose from the University of Lübeck for reading our article and providing *IL-6* and *IL-10* primers as a gift.

## Author contributions

B.L., I.O., and V.P. conducted experiments and analyzed data. V.P. contributed to article drafting and data interpretation and wrote the article. L.S. conducted FACS measurements. S.S., I.H., andK.B. collected tissues for molecular assays. C.D.S. contributed to article drafting, gave advice for experimental design, and provided mice. H.O. designed the research, supervised the project, and contributed to article drafting. All authors reviewed and revised the article.

## Declaration of interests

The authors declare no competing interests.

## STAR★Methods

### Key resources table

**Table:** 

REAGENT or RESOURCE	SOURCE	IDENTIFIER
**Antibodies**		

anti-F4/80	bio-rad	CAT#MCA 497R; RRID:AB_323279
CD163	Abcam	CAT#ab182422; RRID:AB_2753196
CD11c	Abcam	CAT#ab219799; RRID:AB_219799
UCP1	Abcam	CAT#ab209483; RRID:AB_2722676
anti-rabbit Alexa 488	Invitrogen	CAT#A-11008; RRID:AB_143165
anti-rat Alexa 488	Invitrogen	CAT#A-11006; RRID:AB_141373
CD16/CD32 (Fc block)	BD Biosciences	CAT#553141; RRID:AB_394656
CD45-FITC	BioLegend	CAT#103108; RRID:AB_312973
CD11b-PerCP/Cy5.5	BioLegend	CAT#101228; RRID:AB_893232
Ly6G-BV421	BioLegend	CAT#127627; RRID:AB_10897944
F4/80-PE	BioLegend	CAT#123109; RRID:AB_893498
CD206-PE/Cy7	BioLegend	CAT#141720; RRID:AB_2562248
CD11c-APC	BioLegend	CAT#117310; RRID:AB_313779

**Chemicals, peptides, and recombinant proteins**		

SYBR Safe	Invitrogen	CAT#S33102
DAPI, 25 mg	Roth	CAT#6335.1
powdered D(+) glucose	ROTH	CAT#G8270
insulin stock solution	Sigma-Aldrich	CAT#I9278
sodium pentobarbital (Narcoren)	Boehringer Ingelheim	PZN:11336163
DPX mounting medium	Sigma-Aldrich	CAT#06522
Collagenase type I	Gibco	CAT#17100017
NeoClear (xylene substitute)	Sigma-Aldrich	CAT#109843
PBS	FisherScientific	CAT#11503387
Nacl (0.9%)	Braun	CAT#3570310
paraffin	Sigma-Aldrich	CAT#107150
paraformaldehyde (PFA)	Sigma-Aldrich	CAT#158127
100bp DNA ladder	Lonza	CAT#50327
25 000 I.E./5ml heparin	Rotexmedica	PZN:03862340

**Critical commercial assays**		

CD45 MicroBeads	Miltenyi Biotech, Bergisch-Gladbach	CAT#130-052-301
Zombie NIR (APC/Cy7)	BioLegend	CAT#423105
monocytes: Monocyte Isolation Kit	Miltenyi	CAT#130-100-629
Anti-F4/80 MicroBeads UltraPure	Miltenyi	CAT#130-110-443
Anti-Ly-6G MicroBeads, UltraPure	Miltenyi	CAT#130-120-337
Pan Dendritic Cell Isolation Kit	Miltenyi	CAT#130-100-875
mouse adiponectin ELISA kit	Crystal Chem	CAT#80569
High-Capacity cDNA Reverse Transcription Kit	Applied Biosystems, Massachusetts, USA	CAT#4368814
Go-Taq qPCR Master Mix Kit	Promega GmbH, Mannheim, Germany	CAT#A6002
Eosin y solution	Sigma-Aldrich	CAT#HT110216
Hematoxylin	Sigma-Aldrich	CAT#GHS132-1L

**Deposited data**		

Raw and analysed data	This paper	N/A

**Experimental models: Organisms/strains**		

B6.Cg-Tg(S100A8-cre,-EGFP)1Ilw/J	*Mrp8*-*Cre*, Jackson Laboratory	Stock No: 021614
B6.129S4(Cg)-*Arntltm1Weit*/J	*Arntl-*flox or *Bmal1*-flox, Jackson Laboratory	Stock No: 007668

**Oligonucleotides**		

Primers	This paper [Table S2]	N/A
Primer *Eef1α*: 5’- TGCCCCAGGACACA GAGACTTCA - 3’, 5’- AATTCACCAACACCAGCAGCAA-3’	Eurofins	N/A
Primer *Klf4*: 5’- GAAATTCGCCCGCT CCGATGA - 3’, 5’- CTGTGTGTTTGCGGTAGTGCC - 3’	Eurofins	N/A
Primer *Il-10*: 5’- GCTGTCATCGATTT CTCCCC - 3’, Primer 5’- ACACCTTGGTCTTGGAGC TTAT - 3’	Eurofins	N/A
Primer *Tnfα*: 5’- GAAAAGCAGCAGC CAACCA - 3’, 5’- CGGATCATGCTTTCTGTGCTC - 3’	Eurofins	N/A
Primer *Il-6*: 5’- CTCCCAACAGACCT GTCTATAC - 3’, 5’- GTGCATCATCGTTGTTCATAC - 3’	Eurofins	N/A
Primer *Cxcl2*: 5’- GTTTGCCTTGACC CTGAAGCC - 3’, 5’- TCTCAGACAGCGAGGCACAT - 3’	Eurofins	N/A
Primer *Cxcr2*: 5’- ATGCCCTCTATTC TBCCAGAT - 3’, 5’- GTGCTCCGGTTGTATAAGATGAC - 3’	Eurofins	N/A
Primer *Cxcr4*: 5’- GCGTTTGGTGCTCC GGTAAC - 3’, 5’- TTCATCCCGGAAGCAGGGTT - 3’	Eurofins	N/A
Primer *Anxa1*: 5’- CAAAGGTGGTCCT GGGTCAG - 3’, 5’- TTCTCCTGTAAGTACGCGGC - 3’	Eurofins	N/A

**Software and algorithms**		

FIJI/ImageJ	ImageJ	https://imagej.net/software/fiji/
Gen5 software	BioTek Instruments	N/A
Prism 9 software	GraphPad	N/A
ClockLab software	ClockLab version 6.1.02, Actimetrics	https://actimetrics.com/products/clocklab/
circacompare R package v.0.1.1	Parsons et al.^85^	https://doi.org/10.1093/bioinformatics/btz730
JTK_CYLCE	Hughes et al.^86^	https://doi.org/10.1177/0748730410379711
Measure minispec Plus NF	Bruker	N/A

**Other**		

ventilated cages	SealSafe Plus GM 500, Tecniplast	https://www.tecniplast.it/de/product/dgm.html
breeding diet	Altromin	CAT# 1314
open cages	1284 L Eurostandard type II L, Tecniplast	https://www.tecniplast.it/de/product/dgm.html
High fat diet (HFD)	Research Diets	CAT#D12492i
Chow diet (Altromin)	Research Diets	CAT#D12450B
FastGene Blue/Green GelPic LED Box Imaging System	Nippon Genetics	N/A
Microtome	Thermo Scientific HM 340E	https://www.fishersci.com/shop/products/hm-340e-electronic-rotary-microtome/p-4529948
Zeiss Cell Discoverer 7 microscope	Zeiss	https://www.zeiss.com/microscopy/de/produkte/imaging-systeme/celldiscoverer7.html
glucometer	ACCU-CHEK Aviva, Roche	CAT#04455215003
microplate spectrophotometer (Epoch Microplate Spectrophotometer)	BioTek Instruments	N/A
scil Vet abc Plus+ hematology analyser	Scil Animal Care Company	https://www.scilvet.com/products/laboratory-diagnostics/hematology/scil-vet-abc-plus
ClockLab system	ClockLab version 6.1.02, Actimetrics	https://actimetrics.com/products/clocklab/
EDTA-coated microvettes	Sarstedt	CAT# 20.1288
nuclear magnetic resonance	Bruker	minispec LF110
indirect calorimetry system (PhenoMaster)	TSE Systems	N/A
EDTA-coated tubes	Sarstedt	CAT#41.1504.005
Canto II flow cytometry analyzer	BD Biosciences	N/A
MACS Smart Strainers 30 μm	Miltenyi	CAT#130-098-458
MACS Smart Strainers 70 μm	Miltenyi	CAT#130-098-462
Compensation beads	BD Biosciences	CAT#51-90-900 1291
Compensation Beads	BD Biosciences	CAT#51-90-9000 941
FACS	This paper	Cell Analysis Core Facility at the University of Lübeck
iCycler	BioRad, Hercules, California, USA	N/A

### Experimental model and study participant details

#### Animals

Mice with *Bmal1* deficiency in neutrophils (NE-BKO) were generated by crossing B6.Cg-Tg(S100A8-cre,-EGFP)1Ilw/J (*Mrp8*-*Cre*, Jackson Laboratory Stock No: 021614) with B6.129S4(Cg)-*Arntltm1Weit*/J mice (*Arntl-*flox or *Bmal1*-flox, Jackson Laboratory Stock No: 007668). The genetic background of all mice was C57BL/6J. For the experiments male only were used. All animals were kept in the animal facility of the University of Lübeck at room temperature (21 - 24°C), a humidity of 55 - 65 % and a light-dark cycle with 12 h light and 12 h darkness. In all experiments, the time of “lights on” is referred to as *Zeitgeber* time (ZT) 0 and the time of “lights off” as ZT12. Before experiments, mice were housed in specific pathogen-free conditions in groups of up to 5 animals per cage in individually ventilated cages (SealSafe Plus GM 500, Tecniplast). They had *ad-libitum* access to regular chow (breeding diet CAT#1314, Altromin) and tap water. For the experiments, male mice were transferred to the experimental unit. Here, they were kept in open cages (1284 L Eurostandard type II L, Tecniplast) and in light-tight cabinets where light conditions could be set individually. Whenever food intake was monitored (all high-fat diet (HFD) experiments and the corresponding short-term experiments with chow), mice were single-housed so that food intake could be related to a specific animal. All animal experiments were performed according to the German law on animal welfare and the guidelines for animal research by the Federation of European Laboratory Animal Science Associations (FELASA). Before experiments were conducted, the experimental outline was subject to ethical assessment and to legal approval by the “Ministerium für Energiewende, Landwirtschaft, Umwelt, Natur und Digitalisierung” of the state of Schleswig-Holstein. Experiments were performed under license numbers 4(58-5/19), 4(37-4/18), 4_2017-08-30, and 4_2020-09-23.

### Method details

#### PCR to validate Bmal1 deficiency in neutrophils

3 weeks old male mouse (NE-BKO) was sacrificed at ZT 1 to harvest spleen, bone marrow, and brain. To assess *Mrp8-Cre* -mediated recombination, immune cells were isolated from tissues, and genomic DNA was digested with AvrII to facilitate PCR genotyping. The following kits and reagents were used for cell isolation according to the manufacturers' protocols: lymphocytes: CD45 MicroBeads (CAT#130-052-301; Miltenyi Biotech, Bergisch-Gladbach, DE); monocytes: Monocyte Isolation Kit (CAT#130-100-629; Miltenyi); macrophages: Anti-F4/80 MicroBeads UltraPure (CAT#130-110-443; Miltenyi); neutrophils: Anti-Ly-6G MicroBeads, UltraPure (CAT#130-120-337; Miltenyi); dendritic cells: Pan Dendritic Cell Isolation Kit (CAT #130-100-875; Miltenyi). Genomic DNA was extracted by the addition of 20 μl Lysis buffer (50 mM Tris/HCl pH 8.0, 10 mM EDTA pH 8.0, 2 mM NaCl, 1% Sodium dodecyl sulfate). The extracted genomic DNA was used as a template to determine the *Bmal1*-flox genotype as well as the presence of Cre recombinase by polymerase chain reaction (PCR). PCR amplification was carried out with an initial denaturation at 94°C for 3 minutes, followed by 35 cycles of 94°C for 30 seconds, 62°C for 1 minute, and 72°C for 1 minute, with a final extension at 72°C for 5 minutes. PCR products were separated on a 1.5% agarose gel and visualized using SYBR Safe (Invitrogen) and the FastGene Blue/Green GelPic LED Box Imaging System (Nippon Genetics). The following primers were used for PCR: *Bmal1-KO* forward: 5' - CTC CTA ACT TGG TTT TTG TCT GT - 3', *Bmal1-flox* forward: 5' - ACT GGA AGT AAC TTT ATC AAA CTG - 3', *Bmal1-flox* reverse: 5' - CTG ACC AAC TTG CTA ACA ATT A - 3'. *Mrp8-Cre*-mediated recombination with *Bmal1-flox*, resulting in *Bmal1* knockout (KO), was identified by the presence of two bands: a 431 bp band corresponding to the *Bmal1-flox* allele and a 570 bp band corresponding to the *Bmal1-KO* allele. As a size marker a 100bp DNA ladder (CAT#50327; Lonza) was used. In the absence of *Mrp8-Cre* recombination, only the 431 bp *Bmal1-flox* allele band was observed (Figure 1A).

#### Recording of locomotor activity

Locomotor activity was measured in 4-5 weeks old male mice. For the chow experiment, control (n = 4) and NE-BKO (n = 6) mice were single-housed in cages equipped with running wheels (Altromin, 10% fat, CAT# D12450B; Research Diets). For the HFD experiment a new cohort of animals was used consisting of control (n = 16) and NE-BKO (n = 16) mice. These mice were also single-housed, with locomotor activity monitored using infrared detectors installed above the cages. For running-wheel experiments, the number of wheel revolutions per time was recorded. Infrared detectors detected movements in their sensor area as beam breaks per time. Both recordings were analyzed in 5-min intervals using the ClockLab system and software (ClockLab version 6.1.02, Actimetrics).

#### HFD feeding

At the beginning of the experiment all male mice were 4-5 weeks old (control: n = 16, NE-BKO: n = 16). They were single-housed in cages equipped with infrared detectors. Before starting the HFD, they were maintained on a chow diet for one week (Altromin, 10% fat, CAT# D12450B; Research Diets), after which they were switched to HFD, with 60% of calories derived from fat (CAT# D12492i, Research Diets). To assess the effects of short- and long-term HFD on activity and food intake, measurements were taken one week after starting the HFD and continued weekly for 20-23 weeks. Food intake was assessed by weighing the remaining food in the hoppers.

#### Analyses of feeding rhythms

To analyze daily feeding rhythms in control and NE-BKO male mice, food hoppers of single-housed animals were weighed at 6-hour intervals over a 30-hour period. To minimize disturbance, both time and noise during measurements were kept to a minimum. When food was weighed during the dark phase, red light was used to avoid disrupting the circadian system.

For this for this purpose, two separate cohorts of male mice were used. One cohort consisted of chow-fed male mice (control: n = 4, NE-BKO: n = 6), which were 18-23 weeks old when food profiles were recorded. The second cohort consisted of male mice that received HFD. During HFD experiments, food profiles were recorded for control (n = 16) and NE-BKO (n = 16) male mice at two time points: one week after the start of HFD feeding (age 5–6 weeks) and again towards the end of the experiment (17–20 weeks after the start of HFD).

#### Glucose tolerance tests

Glucose tolerance tests were performed in a randomized order on control and NE-BKO male mice (n = 16/16) during long-term HFD feeding experiments, 15-16 weeks after start of HFD feeding. Tests were performed around ZT3 (± 30 min). Food was removed 16 h prior to the test so that animals were in a fasted state during the test. Initial blood glucose levels were determined by taking a drop of blood from the tail vein and measuring glucose concentration using a glucometer (CAT# 04455215003, ACCU-CHEK Aviva, Roche). Next, glucose solution was injected intraperitoneally (1.5 g/kg body weight, powdered D(+) glucose (CAT#G8270, ROTH) in 0.9 % NaCl (CAT# 3570310, Braun). Blood glucose measurements were repeated 15, 30, 60 and 120 min after the injection. At the end of the tests, animals were provided with food *ad libitum*.

#### Insulin tolerance tests

Since glucose and insulin tolerance tests both require repeated withdrawal of blood, the same male mice used for glucose tolerance test were given one week of recovery between both tests. Therefore, insulin tolerance tests were performed 16-17 weeks after start of HFD feeding. The tests were performed in a similar way as the glucose tolerance tests, but food was removed only 4 h prior to the start. Insulin was injected at a concentration of 1 U/kg body weight. Human insulin stock solution (CAT#I9278, Sigma-Aldrich) was diluted with 0.9 % NaCl (CAT# 3570310, Braun).

#### Adiponectin measurement

Two new groups of male mice (control and NE-BKO, n = 15/16 each) were single-housed in cages equipped with infrared detectors. One group (n = 15/16) was euthanized one week after starting the HFD at ZT1. For ventricular plasma collection, mice were first deeply anaesthetised by intraperitoneal injection of sodium pentobarbital (PZN:11336163, Narcoren, Boehringer Ingelheim, 320 mg/kg body weight). The injected solution also contained 1,000 U/ml sodium heparin (PZN:03862340, Rotexmedica, 10,000 U/kg body weight were injected) to prevent blood clotting. After careful assessment of negative toe pinch reflexes, the chest was opened. Blood was withdrawn from the right ventricle using an insulin syringe (BD Biosciences). Blood samples were immediately transferred to EDTA-coated tubes (CAT#41.1504.005, Sarstedt), which were kept on ice until centrifugation at 4°C, 664 x g for 15 min. Supernatants (plasma) were frozen at -80°C until further use. Following blood collection, the circulation of the animals was perfused with 10 ml phosphate-buffered saline (PBS, Gibco, CAT#11503387, FisherScientific). After blood collection, the mice were decapitated. The second group was euthanized at ZT1 after 20 weeks on the HFD to collect plasma. Plasma adiponectin levels were determined using sandwich enzyme-linked immunosorbent assay (ELISA) kit (CAT#80569, mouse adiponectin ELISA kit, Crystal Chem) according to the manufacturer's instructions. Briefly, adiponectin was captured by a specific antibody coated on a microplate. A second specific antibody was then added to bind to the immobilised adiponectin. The second antibody was biotinylated. The addition of the substrate solution initiated a colorimetric response that could be detected on a microplate spectrophotometer (450/630 nm, Epoch Microplate Spectrophotometer, BioTek Instruments) in combination with Gen5 software (BioTek Instruments). Plasma concentrations were interpolated from standard curves using Prism 9 software (GraphPad).

#### Tissue collection for neutrophil and macrophage analysis

Mice used for plasma collection to determine adiponectin after one week and 20 weeks of HFD were decapitated after blood collection. After decapitation, eWAT fat pads and inguinal subcutaneous (sc)WAT depots were collected. Adipose tissues were weighed and the lymph nodes of scWAT excised. One eWAT and one scWAT fat pad were separately cut into small pieces and placed into ice-cold adipose tissue dissociation buffer for isolation of immune cells.

#### Isolation of immune cells from adipose tissue

An abundance of immune cells, i.e., neutrophils, and macrophages, in adipose tissue was estimated. Adipose tissue depots were cut into small pieces and placed in adipose tissue dissociation buffer containing 100 mM HEPES pH 7.4, 120 mM NaCl, 50 mM KCl, 5 mM glucose, 1 mM CaCl_2_, 1.5 % bovine serum albumin (BSA) and 1 mg/ml collagenase (CAT#17100017, Collagenase type I, Gibco). Pieces of adipose tissue were placed into conical tubes with 9 ml dissociation buffer and incubated at 37°C for 45 min, shaking at 250 rpm (Ecotron Incubator Shaker, Infors HT). The cell suspensions were then passed through 70-μm MACS Smart strainers (CAT#130-098-462, Miltenyi) and the flowthroughs were incubated at room temperature for 10 min. During that time, floating adipocyte fractions separated from the stromal vascular fractions below. Stromal vascular fractions were collected with a pipette and transferred to fresh tubes for centrifugation (400 x g, 10 min, 4°C). Supernatants were decanted, and the inverted tubes were dried on a paper towel. Pellets were then dissolved in 1 ml pre-chilled RPMI medium and transferred to 1.5-ml tubes. Cells were counted and up to 3×10^6^ cells were stained for flow cytometry analysis.^87^

#### Staining of adipose tissue-derived cells for flow cytometry

All centrifugation steps in this procedure were performed at 300 x g for 5 min at room temperature. Tubes were filled with PBS, centrifuged, and the pellets were resuspended in 100 μl PBS. Next, 4.0 μl Zombie NIR, diluted 1:100 in PBS (see Table S1) was added and incubated for 15-30 min at room temperature in the dark. The cells were then washed with 1 ml MACS buffer (PBS containing 2 mM EDTA and 0.5% BSA, pH 7.2) and centrifuged again. Subsequently, pellets were resuspended in 100 μl MACS buffer and 1.2 μl antibodies against Fcγ receptor (FCGR) 3 (CD16) and FCGR2 (CD32) were added (Fc block, diluted 1:10 in PBS; see Table S1) and incubated at 4°C for 5 minutes. After incubation, antibodies against protein tyrosine phosphatase, receptor type, C (PTPRC, CD45), lymphocyte antigen 6 complex locus 6GD (Ly6G), integrin subunit α M (ITGM, CD11b), adhesion G protein-coupled receptor E1 (ADGRE1, F4/80), integrin subunit alpha X (ITGAX, CD11c) and macrophage mannose receptor (MMR, CD206) were added directly to the cell suspensions (see Table S1). These antibodies were coupled to the fluorochromes fluorescein (FITC), brilliant violet 421 (BV421), peridinin chlorophyll protein - cyanine 5.5 (PerCP/Cy5), phycoerythrin (PE), APC and PE - cyanine 7 (PE/Cy7) as specified below.2.0 μL CD45-FITC (diluted 1:20 in PBS), 1,5 μL Ly6G-BV421. 0.7 μl CD11b-PerCP/Cy5.5, 1.75 μl F4/80-PE, 0.7 μl CD11c-APC, 4 μl CD206-PE/Cy7 (diluted 1:10 in PBS). These antibodies were then incubated at 4°C for 15-30 min. The samples were then washed with 1 ml MACS buffer to remove unbound antibodies, centrifuged again, and the pellets resuspended in 300 μl paraformaldehyde (1 % in PBS) to fix the cells until fluorescence-activated cell sorting (FACS) measurements were performed the following day.

#### Flow cytometry analysis

Samples that were prepared as described above were transferred to 5 ml flow cytometry tubes on the day of measurements. Immune cells were assessed using an LSR II or Canto II flow cytometry analyzer (BD Biosciences). The cytometers were compensated using single-stained compensation microbeads in combination with negative control beads (CAT#51-90-900 1291 and CAT#51-90-9000 941, BD Biosciences). For an equal proportion of live and dead cells, splenocytes were incubated at 60°C for 5-10 min and then immediately placed on ice. The staining allowed quantification of neutrophils and macrophages, with macrophages further subdivided into M1-like and M2-like macrophages (see Figure S1). A dump channel was included to manage the high degree of autofluorescence that is typical of adipose tissue cells. Isotype controls were used to exclude unspecific binding of the antibodies. Fluorescence-minus-one controls were used to ensure proper gate setting. In addition, replicates of unstained control samples were measured in each lot.

#### Blood cell count

To determine blood cell counts of red blood cells (RBC), platelets (PLT), and white blood cells (WBC) in 4-5 weeks old male mice under chow condition, control and NE-BKO mice (n = 6 each) were euthanized at ZT1 (±30 minutes) via cervical dislocation followed by decapitation. Trunk blood was collected into EDTA-coated microvettes (CAT#20.1288, Sarstedt) and analyzed using the scil Vet abc Plus+ hematology analyser (Scil Animal Care Company).

#### Body composition

A new group of 4-5 weeks old male mice (control: n = 12, NE-BKO: n = 11) was used for the HFD experiment . The body composition of the animals was analysed by nuclear magnetic resonance (minispec LF110, Bruker). Measurements were performed during week 16-17 of HFD feeding around ZT9 (± 30 min). Mice were immobilized in the measurement tube for a total time of 2 min. During that time, absolute amounts of free body fluid, fat mass, and lean mass were detected. Data were processed using the manufacturer’s software (Measure minispec Plus NF, Bruker).

#### Indirect calorimetry

Oxygen consumption (VO_2_) and carbon dioxide production (VCO_2_) were analysed using an open-circuit indirect calorimetry system (PhenoMaster, TSE Systems). For the experiments new group of 4-5 weeks old male mice (control: n = 12, NE-BKO: n = 12) was used for the HFD experiment. Mice were performed 14-16 weeks after the start of the HFD. One week before the start of the measurements, the mice were acclimatised to the water bottles used in the calorimetry system. Respirometry data collected at 20-minute intervals were used to assess energy expenditure.^88^^,^^89^^,^^90^ Energy expenditure was assessed from day 3 of the measurement. Three consecutive data points were averaged to generate data with a time resolution of one hour. Data on oxygen consumption and respiratory exchange ratio (RER= VO_2_/VCO_2_) were further used to calculate energy expenditure^90^: *Energy expenditure* [*kJ*ℎ]= (4.44+1.43∗*RER*)∗VO_2_∗0.0036. Only the last day of measurement was used for analysis. For one cohort, the calorimetry device recorded oxygen consumption and carbon dioxide production for only half of the channels. Therefore, measurements of four Control and two NE-BKO animals had to be terminated prematurely. Here, the fourth day was used for analysis. The calorimetry system collected data in 20-min intervals. Three consecutive data points were averaged to generate data with a time resolution of one hour.

#### Tissue sectioning for histology

A group of male animals (4-5 weeks old) consisting of control (n = 5) and NE-BKO (n = 6) mice was used. These mice were singly housed and maintained on HFD for 20 weeks. After 20 weeks of HFD adipose tissue, e.g., eWAT and iBAT, was harvested from mice. Mice were euthanized by cervical dislocation followed by head decapitation. Tissue samples were initially fixed in 4% paraformaldehyde (PFA) (CAT#158127, Sigma-Aldrich) at 4°C for 24 hours. After fixation, the tissue was sequentially transferred to 30% ethanol (EtOH) for 6 hours, followed by 50% EtOH for 6 hours, and then 70% EtOH for 12 hours, all at 4°C. The tissue was then stored in 100% EtOH at -20°C. For embedding, the tissue was first immersed in a 50% EtOH / 50% xylene solution at room temperature for 1 hour, followed by a 1-hour transfer to 100% xylene (NeoClear (xylene substitute), CAT#109843, Sigma-Aldrich). Next, the tissue was placed in a 50% xylene / 50% paraffin (CAT#107150, Sigma-Aldrich) solution at 58°C for 1 hour, followed by three 1-hour transfers to 100% paraffin at 58°C. Finally, the tissue was embedded in paraffin within a tray and allowed to harden at room temperature. Tissue sections were cut to a thickness of 5 μm using a microtome (Thermo Scientific HM 340E) and collected in a 34°C warm water bath. Sections were then transferred onto glass slides and allowed to air-dry at room temperature. To deparaffinize, sections were treated with two changes of 100% xylene for 5 minutes each, followed by two changes of 100% ethanol for 3 minutes each, and then 95% ethanol and 80% ethanol for 1 minute each. After rinsing with distilled water, antigen retrieval was performed to facilitate immunofluorescence staining. Antigen retrieval was carried out in 10 mM sodium citrate buffer (pH 6.0) at 95°C for 20 minutes. After cooling for 20 minutes, the sections were rinsed with phosphate-buffered saline (PBS 1X) three times for 10 minutes each before starting with immunofluorescence staining.

#### Hematoxylin and eosin (H&E) staining of eWAT and iBAT

The eWAT and iBAT tissues, as described in the 'tissue sectioning for histology' section, were subjected to morphological analysis using H&E staining or immunofluorescence. Sections were deparaffinized in NeoClear (CAT# 109843, xylene substitute; Sigma-Aldrich) and rehydrated through a graded ethanol series: 100% ethanol (2 changes, 5-10 minutes each) 95% ethanol (5-10 minutes) 70% ethanol (5-10 minutes) distilled water (5-10 minutes). Staining began with a 2- minutes. immersion in hematoxylin, followed by 10 minutes of blueing under running tap water. Differentiation was performed in acidic 70% ethanol, and sections were subsequently stained with eosin for 4 minutes. Dehydration was completed through an ascending ethanol series, and sections were mounted using DPX mounting medium (CAT#06522, Sigma-Aldrich). Imaging and Analysis: H&E-stained sections were visualized under brightfield with contrast using a Nikon light microscope. For eWAT, images were captured at 20X magnification. For iBAT, both 20X and 40X magnifications were used, depending on the sample characteristics. Specifically, 40X magnification was employed for droplet size measurements, except in two control group samples where large droplet sizes necessitated the use of 20X magnification. Lipid droplet sizes were quantified using ImageJ software. For each image, 10 lipid droplets were measured, beginning with an arbitrarily selected droplet at the center of the image. Subsequent measurements proceeded in a clockwise direction to neighbouring droplets.

#### Immunofluorescence staining of eWAT and iBAT

For immunofluorescence staining, the tissue sections containing eWAT or iBAT tissue were rinsed three times with 1X PBS for 10 minutes each. The sections were then blocked with 5% goat serum in PBST (1X PBS containing 0.1% Tween-20 and 0.5% Triton X-100) for 1 hour at room temperature. Following blocking, eWAT sections were incubated overnight at 4°C with primary antibodies targeting macrophage markers: 1:200 anti-F4/80 (marker for macrophages, MCA 497R bio-rad), 1:200 CD163 (marker for macrophage type 2, ab182422 Abcam), or 1:200 CD11c (marker for macrophage type 1, ab219799 Abcam). For iBAT sections, 1:200 UCP1 antibody (ab209483 Abcam) was used as the primary antibody. The next day, slides were washed three times with 1X PBS for 10 minutes each. Subsequently, sections were incubated with the secondary antibody (CAT#A-11008, 1:500 anti-rabbit Alexa 488; Invitrogen, or 1:500 anti-rat Alexa 488; CAT#A-11006, Invitrogen) for 2 hours at room temperature. After secondary antibody incubation, the slides were washed again three times with 1X PBS and mounted using a DAPI-containing mounting medium (CAT#6335.1 ROTH) (final DAPI concentration: 5 μg/ml). For each genotype, tissues from seven animals were analysed. Images were captured using the Zeiss Cell Discoverer 7 microscope at 20X magnification. Negative control sections incubated only with the secondary antibody were used to determine and exclude nonspecific staining and background fluorescence. Fluorescence intensity was quantified using the FIJI/ImageJ software. The intensity values were normalized to the area of the tissue and scaled to the maximum fluorescence intensity (100%).

#### Real-time PCR

Gene expression was analyzed in the tissues of two cohorts of male mice during both the light and dark phases, with the mice kept in individual cages without running wheel equipment. The first cohort consisted of control (n = 26) and NE-BKO (n = 25) mice, which were maintained on HFD for 20 weeks. On the 20th week, these mice were euthanized for tissue collection. The second cohort consisted of control (n = 10) and NE-BKO (n = 10) mice that were kept on a chow diet for two weeks. This chow-fed cohort was age-matched to the HFD cohort, meaning that all mice were approximately 23–24 weeks old at the end of the experiment. eWAT was collected from both animal groups under the LD cycle. At the end of each experiment (HFD and chow), mice were euthanized during the light phase (ZT1 and ZT7) or the dark phase (ZT13 and ZT19) via intraperitoneal injection of sodium pentobarbital (PZN: 11336163 Narcoren, Boehringer Ingelheim, 320 mg/kg body weight) to ensure deep anesthesia. If euthanasia was performed during the dark phase, all procedures were carried out under dim red light to minimize disruption of the circadian rhythm. eWAT fat pads were meticulously excised anterior to the epididymis, followed by immediate snap-freezing of tissue samples using dry ice. The RNA of the homogenised eWAT tissue was isolated and purified from the homogenate. For qPCR measurement, 300 ng of RNA per sample was transcribed into cDNA; reverse transcription was performed using the High-Capacity cDNA Reverse Transcription Kit (CAT#4368814, Applied Biosystems, Massachusetts, USA). Quantitative real-time PCR was performed on an iCycler (BioRad, Hercules, California, USA) using the Go-Taq qPCR Master Mix Kit (CAT#A6002, Promega GmbH, Mannheim, Germany) according to the manufacturer's protocol. Samples were run in duplicates. For the estimation of single-well amplification efficiency and relative quantification of expression levels, we employed a linear regression analysis on the logarithm of fluorescence data per cycle number. This method allows for an assumption-free calculation of starting concentrations and PCR efficiencies for each sample, enhancing the accuracy of quantification. The analysis was conducted using a Microsoft Excel spreadsheet designed for this purpose, which filters data points to establish a window of linearity and calculates the PCR efficiency based on the slope of the regression line.^91^ Gene expression of *Tnfα*, *Cxcl2*, *Cxcr2*, *Cxcr4*, Kruppel-like factor 4 (*Klf4*), *Anxa1*, *Il-6*, and *Il-10* was analysed with eukaryotic elongation factor-1 α (*eEF1α*) as the housekeeping gene.^92^ The sequences of used primers are provided in (see Table S2). Relative gene expression was subsequently normalised to the control animal group when demonstrating the relative RNA expression of whole-day RNA expression. When comparing RNA expression between day and night, data were normalised to the control during the day phase.

### Quantification and statistical analysis

Statistical tests were performed using GraphPad Prism 9. All data sets were tested for normal distribution using Shapiro-Wilk test. In borderline cases (*p* = 0.05 – 0.04), the QQ-plot was assessed. If also the QQ-plot showed clear deviation from normal distribution, or the p-value was highly significant, data were base-10 log-transformed. Subsequently, for two-factor comparisons, two-way ANOVAs with Tukey’s post-tests were performed. The Kruskal-Wallis-ANOVA test and Wilcoxon test were used to analyse non-normally distributed data. The outliers were checked using the ROUT method (GraphPad Prism) based on non-linear regression. A false discovery rate of 1 % was set, and outliers were removed from the analysis.^93^ Data with similar SDs were analyzed using ordinary two-way ANOVA with Tukey’s post-test. To compare differences between two groups, t-tests were used. Data with similar SDs were analyzed using ordinary one-way ANOVA with Bonferroni post-test. Rhythmic gene regulation was assessed using JTK_CYLCE and a p-value cut-off of 0.05.^86^ Comparison of rhythmic numbers of eWAT neutrophils between NE-BKO and control mice was carried out using circacompare R package v.0.1.1.^85^ P-values below 0.05 were considered significant. The data are presented as means ± SEM, with statistically significant differences indicated by ∗ p ≤ 0.05, ∗∗p ≤ 0.01, ∗∗∗ p ≤ 0.001, ∗∗∗∗ p ≤ 0.0001, ns: not significant. Each specific statistical test used is reported for each experiment in the figure legends.
