# Supplementary material for: Bmal1 deficiency in neutrophils alleviates symptoms induced by high-fat diet
Source: iScience. 2025 Feb 25;28(3):112038. doi: 10.1016/j.isci.2025.112038 (PMC11930374; doi:10.1016/j.isci.2025.112038)
Supplement: Document S1. Figures S1–S8 and Tables S1 and S2 [file mmc1.pdf]

**iScience, Volume *28***

## Supplemental information

# *Bmal1* deficiency in neutrophils alleviates symptoms induced by high-fat diet

**Brinja Leinweber, Violetta Pilorz, Iwona Olejniczak, Ludmila Skrum, Kimberly Begemann, Isabel Heyde, Sarah Stenger, Christian David Sadik, and Henrik Oster**

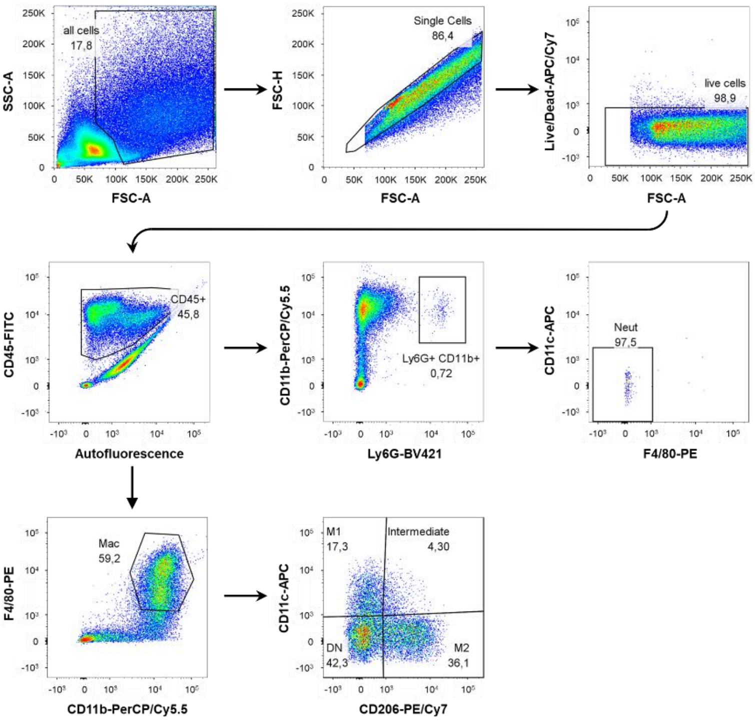


**Figure S1: Gating strategy used for quantification of neutrophils, and macrophages, related to STAR Methods**. First, the cells of interest were separated from debris and dead cells in a forward scatter (FSC) – sideward scatter (SSC) panel. Following this, duplets were excluded in an FSC-FSC panel, and live cells identified as being negative for the live/dead stain. From this population, CD45 positive cells (white blood cells (WBCs)) were plotted against autofluorescence. Cells were further categorized into neutrophils (Neut, positive for CD11b and Ly6G) and macrophages (Mac, positive for F4/80 and CD11b). The final neutrophil population was identified by negative selection for F4/80 and CD11c. Macrophages were further categorized into M1-like (CD11c positive, CD206 negative) or M2-like (CD206 positive, CD11c negative) cells. Macrophages that expressed neither CD11c nor CD206 were classified as double negative (DN).

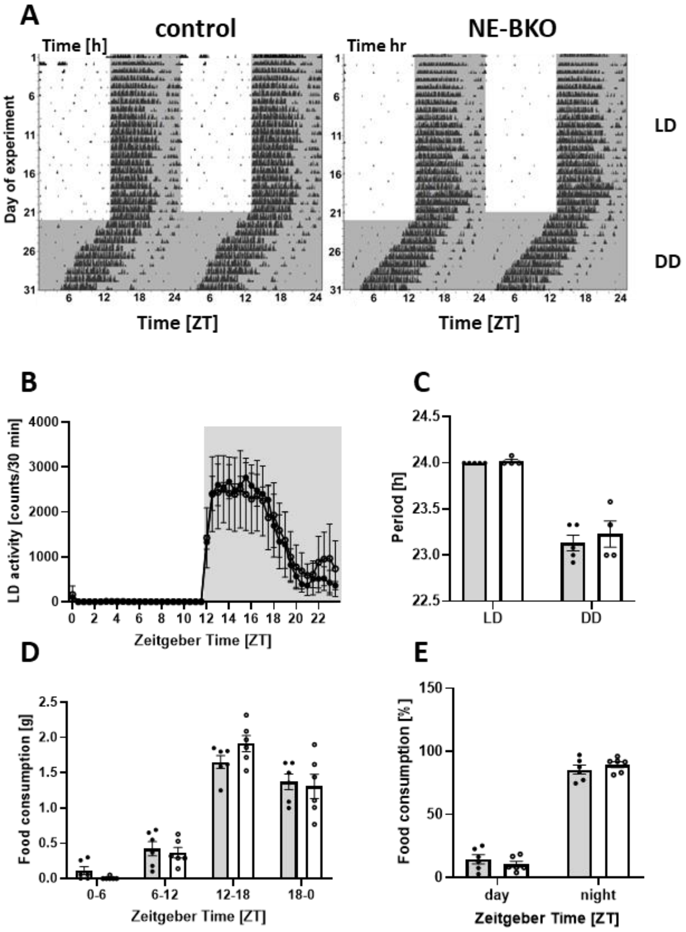


**Figure S2: Wheel-running activity and food intake rhythms are comparable between neutrophil *Bmal1*-KO and control mice, related to Figure 1**. (**A**) Representative double-plotted actograms of a control and an NE-BKO mouse. (**B**) Wheel-running activity profile averaged across 7 days under a light-dark cycle (LD). (**C**) Activity period analysed across 8-17 days in LD and 6-9 days in constant darkness (DD). (**D**) Relative food consumption in LD during a full 24-hour cycle (**E**), and food consumption during day and night across 7 days in LD are similar between control and NE-BKO mice. Grey-shaded areas indicate dark phases. Gray bar: control mice; white bar: NE-BKO mice. Data are presented as means ± SEM, n = 4/6. Significant differences between groups and time were analysed using two-way ANOVA.

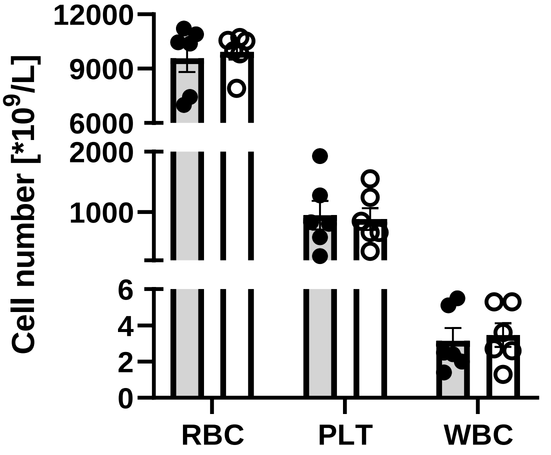


**Figure S3: Unchanged blood cell numbers in neutrophil-specific *Bmal1* deficient mice**. Absolute cell numbers of red blood cells (RBC), platelets (PLT) and white blood cells (WBC) at *Zeitgeber* time (ZT), i.e., 1hr after “lights on”, do not differ between control and NE-BKO mice. Gray bar: control mice, white bar NE-BKO mice. Data are presented as means ± SEMs, n = 6. Significant differences between groups and time were analysed using two-way ANOVA.

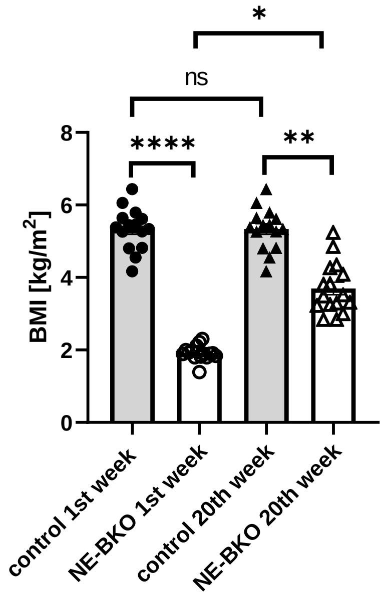


**Figure S4: BMI changes in NE-BKO mice during HFD related to Figure 2.** The BMI of NE-BKO mice undergoes significant changes during the first and the 20th week of HFD, in contrast to control mice whose BMI remains unchanged during these time points. Specifically, in week 20 the BMI of NE-BKO mice is significantly higher than that in the first week of HFD, but significantly lower than that of control mice. Black circles: control mice, $1^{st}$ week of HFD, open circles: first week of HFD, NE-BKO mice, black triangle: $20^{th}$ week of HFD, control mice, open triangle: NE-BKO mice, $20^{th}$ week of HFD. Data are presented as means ± SEM, n = 15/16. Significant differences assessed using Kruskal-Walis ANOVA test are indicated by **** $p \leq 0.0001$, ** $p \leq 0.01$, *$p \leq 0.05$, ns: not significant.

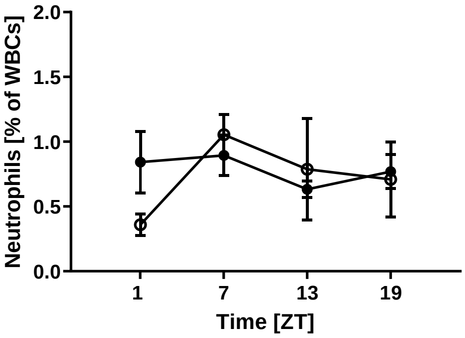


**Figure S5: Neutrophil daily profiles in NE-BKO mice related to Figure 5.** Numbers of neutrophils as percentage of white blood cells (WBCs) in eWAT, n = 7/8 per condition. Tissues were harvested at the end of long-term HFD feeding. Open circles: NE-BKO mice, black circles: control mice. Data are presented as means ± SEM.

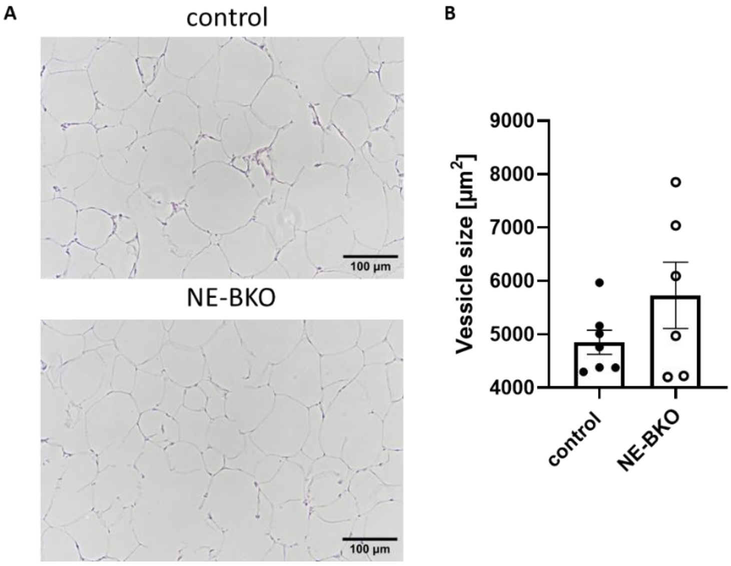


**Figure S6: Lipid Vesicle Size in eWAT After Chronic HFD related to Figure 7.** (**A**) Representative H&E-stained images of eWAT tissue from control and NE-BKO mice. (**B**) After 30 weeks of HFD, both genotypes exhibit similar lipid vesicle sizes (U-test, NE-BKO *vs*. control: U = 8, p = 0.623, n = 6/7). Open circles represent NE-BKO mice, and black circles represent control mice. Data are presented as means ± SEM.

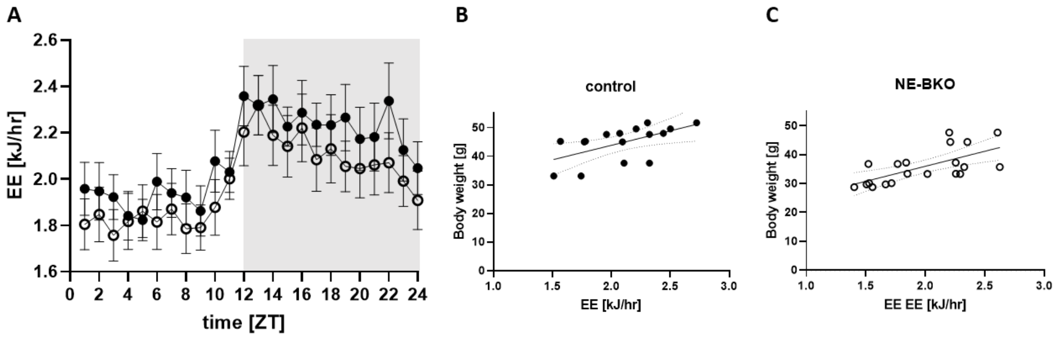


**Figure S7: Effect of Bmal1 deficiency in neutrophils on body weight and energy expenditure during chronic HFD related to Figure 3.**
(**A**) Non-normalized energy expenditure (EE) in control and NE-BKO mice after chronic HFD. (**B-C**) Simple linear regression lines representing the positive correlation between EE and body weight in both control and NE-BKO mice. The correlation is statistically significant in both genotypes (Spearman correlation: control: r = 0.6331, p = 0.010; NE-BKO: r = 0.629, p = 0.004). Open circles represent NE-BKO mice, and black circles represent control mice. Data are presented as means ± SEM.

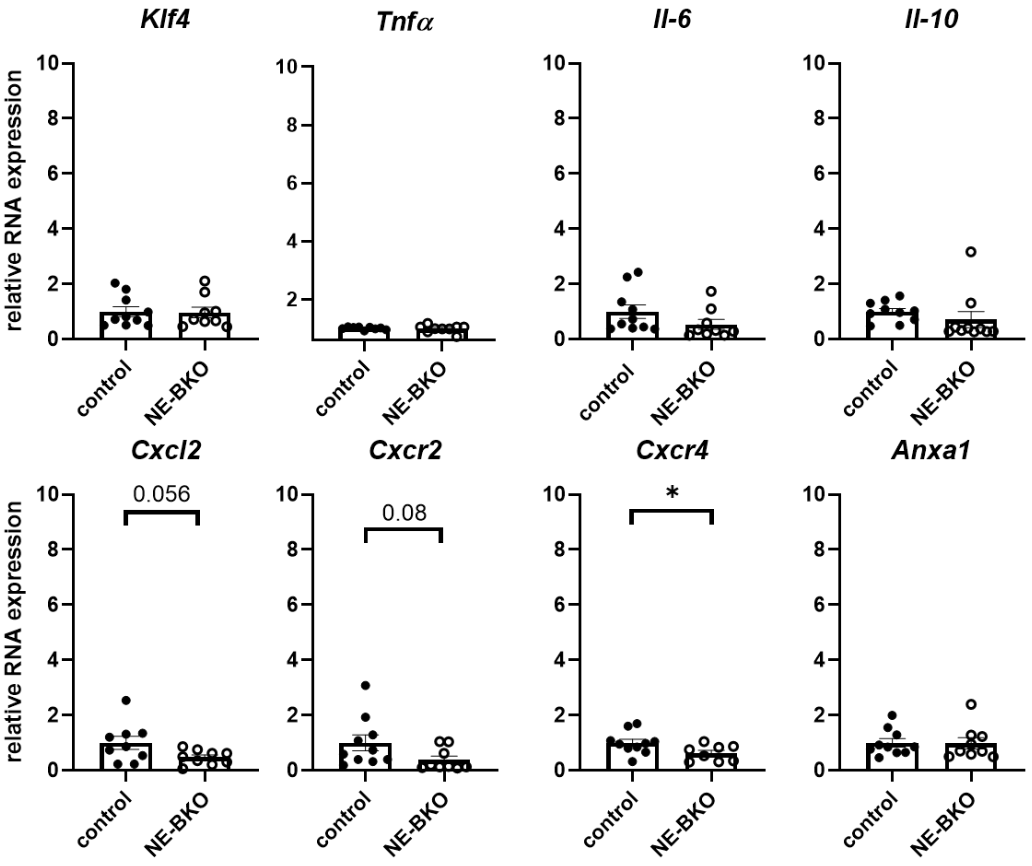


**Figure S8: Effect of Bmal1 deficiency in neutrophils on gene expression in eWAT during a chow diet related to Figure 8 and Figure 9.** Gene expression of pro- and anti-inflammatory factors is comparable between control and NE-BKO mice. However, genes encoding the chemokine Cxcl2 and the chemokine receptors CXCR2 and CXCR4 exhibit a trend toward decreased expression in NE-BKO mice compared to controls (t-test, control vs. NE-BKO, *Cxcl2*: t = 2.060, df = 16, p = 0.056; *Cxcr2*: t = 1.863, df = 17, p = 0.08, n = 9/10), with a significant reduction in Cxcr4 expression in NE-BKO mice relative to controls (t-test, control vs. NE-BKO, *Cxcr4*: t = 2.200, df = 16, p = 0.043, n = 9/10). Filled circles: control mice, open circles: NE-BKO. Data are presented as means ± SEM. Significant differences between both groups (NE-BKO and control) were assessed using t-test, * $p \leq 0.05$.

| Name of the product/antibody | Isotype | Clone | Company | Target cell/purpose |
|---|---|---|---|---|
| Zombie NIR (APC/Cy7) | - | - | BioLegend | Dead cells |
| CD16/CD32 (Fc block) | - | - | BD Biosciences | Block unspecific binding |
| CD45-FITC | Rat IgG2b, κ | 30-F11 | BioLegend | White blood cells (WBCs) |
| CD11b-PerCP/Cy5.5 | Rat IgG2b, κ | M1/70 | BioLegend | Neutrophils and macrophages |
| Ly6G-BV421 | Rat IgG2a, κ | 1A8 | BioLegend | Neutrophils |
| F4/80-PE | Rat IgG2a, κ | BMS | BioLegend | Macrophages |
| CD11c-APC | Armenian hamster | N418 | BioLegend | M1-like macrophages |
| CD206-PE/Cy7 | Rat IgG2a, κ | C068C2 | BioLegend | M2-like macrophages |

**Table S1:** Details of products and antibodies that were used for FACS analysis related to STAR Methods

| | Forward primer (5'→3') | Reverse primer (5'→3') |
|---|---|---|
| *Eef1α* | TGCCCCAGGACACAGAGACTTCA | AATTCACCAACACCAGCAGCAA |
| *Klf4* | GAAATTCGCCCGCTCCGATGA | CTGTGTGTTTGCGGTAGTGCC |
| *Il-10* | GCTGTCATCGATTTCTCCCC | ACACCTTGGTCTTGGAGCTTAT |
| *Tnfα* | GAAAAGCAGCAGCCAACCA | CGGATCATGCTTTCTGTGCTC |
| *Il-6* | CTCCCAACAGACCTGTCTATAC | GTGCATCATCGTTGTTCATAC |
| *Cxcl2* | GTTTGCCTTGACCCTGAAGCC | TCTCAGACAGCGAGGCACAT |
| *Cxcr2* | ATGCCCTCTATTCTBCCAGAT | GTGCTCCGGTTGTATAAGATGAC |
| *Cxcr4* | GCGTTTGGTGCTCCGGTAAC | TTCATCCCGGAAGCAGGGTT |
| *Anxa1* | CAAAGGTGGTCCTGGGTCAG | TTCTCCTGTAAGTACGCGGC |

**Table S2:** Sequences of primers that were used for qPCR related to STAR Methods.
